# Extracellular vesicles derived from astrocyte-treated with haFGF_14-154_ attenuate Alzheimer phenotype in AD mice

**DOI:** 10.7150/thno.70951

**Published:** 2022-05-09

**Authors:** Dong Peng, Youjin Wang, Yuanjie Xiao, Mengyuan Peng, Wanwen Mai, Bo Hu, Yanbin Jia, Hongxia Chen, Yan Yang, Qi Xiang, Zhijian Su, Qihao Zhang, Yadong Huang

**Affiliations:** 1 Department of Cell Biology & Institute of Biomedicine, College of Life Science and Technology, Jinan University, Guangzhou 510632, China.; 2 Guangdong Provincial Key Laboratory of Bioengineering Medicine, Jinan University, Guangzhou 510632, China.; 3 Guangzhou Biopharmaceutical R&D Center of Jinan University Co., Ltd, Guangzhou 510632, China.; 4 Department of Laboratory Medicine, Third Affiliated Hospital of Sun Yat-sen University, Guangzhou 510630, China.; 5 Department of Psychiatry, First Affiliated Hospital of Jinan University, Guangzhou 510630, China.

**Keywords:** Alzheimer's disease, aFGF, AEVs, miR-206-3p, BDNF

## Abstract

**Background:** aFGF content in serum and cerebrospinal fluid is increased in Alzheimer's disease (AD) patients and attenuates the activation of astrocytes. Extracellular vesicles (EVs) are a major mediator in astrocyte-neuron communications. Since excessive or persistent reactive astrocytes lead to chronic inflammation and neuronal dysfunction, and the activation of astrocytes can be inhibited by aFGF, we proposed that the cargoes of astrocyte-derived EVs (AEVs) might be modified by aFGF stimulation, playing an important role in AD progression. However, the mechanisms underlying the role of aFGF remain unclear.

**Methods:** AEVs were isolated from damaged astrocytes, treated with or without aFGF in Aβ-loading condition, and were intranasally administered to AD mice. We determined the ability of AEVs to enter the brain, ameliorate cognitive behavior deficits, alleviate the Aβ burden in the brain, and improve synapse ultrastructure. Subsequently, the miRNAs enriched in AEVs were sequenced to identify the key molecules specifically modified by aFGF. Finally, we explored the protective effects of miR-206-3p inhibition on cognitive deficiency and its regulatory mechanism and determined its role as a specific biomarker for potential AD diagnosis.

**Results:** AEVs stimulated by aFGF (defined as AEVs-_Aβ+H_) had favorable neuroprotection in AD pathology by enhancing neurite growth and reduction of Aβ loading on neurons *in vitro*. Following intranasal administration, AEVs-_Aβ+H_ ameliorated cognitive behavior deficits, promoted synaptic plasticity, and alleviated brain Aβ burden in the APP/PS1 and Aβ brain-injected mice. AEVs-_Aβ+H_ showed beneficial effects on AD similar to AEVs produced in normal situations (AEVs-_Ctrl_). aFGF stimulation modified the cargoes in EVs derived from Aβ damaged astrocytes, the most significant of which being the down-regulation of miR-206-3p. The miR-206-3p level was specifically high in the plasma of AD mice and patients, and miR-206-3p antagomir reversed the Alzheimer phenotype in AD mice. The brain-derived neurotrophic factor (BDNF) gene was negatively regulated by miR-206-3p and upregulated by AEVs_-Aβ+H_ and miR-206-3p antagomir in AD mice. AEVs_-Aβ+H_ inhibited δ-secretase (Asparagine endopeptidase, AEP) activation *via* the miR-206-3p/BDNF axis to alleviate Aβ burden in the AD brain.

**Conclusion:** Our findings highlight the role of aFGF in the modification of AEVs cargoes, especially miR-206-3p that can potentially serve as a biomarker for AD diagnosis and therapeutic target.

## Introduction

Alzheimer's disease (AD) is the most common form of dementia. The pathological hallmarks of the AD brain are senile plaques, neurofibrillary tangles (NFTs), glial dysfunction, and neuronal loss 1-3. There is currently no curative medication for the disease because of its complex pathology. For many years, drug discovery has focused on protecting degenerative neurons. However, increasing evidence has indicated that the astrocyte-neuron network is crucial to the pathogenesis of AD. Astrocytes constitute a large proportion of the cell population in the central nervous system (CNS). Drug discovery efforts targeting astrocytes have attracted much attention in the past decade 4. It has been reported that Aβ oligomers induce astrocytic over-activation, disturb intracellular calcium homeostasis, impair mitochondrial function, aggravate the production of reactive oxygen and nitrogen species, and increase the release of toxic glutamine and proinflammatory factors 3, 5. A series of events in the reactive astrocytes eventually lead to synapse loss and neuron death. Therefore, astrocytes may be a target to attenuate the pathological progression of AD and provide a novel strategy for AD treatment.

Extracellular vesicles (EVs) are a major mediator in astrocyte-neuron communication. The EVs secreted by astrocytes contain many active molecules, such as proteins, lipids, mRNA, and miRNA, which play an important regulatory role in the physiological and pathological processes of the central nervous system. Astrocyte-derived EVs (AEVs) activated by IL-1β and TNF-α inhibit neurite outgrowth in the AD brain 6, 7. Astrocytes also release toxic proteins such as Aβ, ApoE ɛ4, ceramide, and PAR-4 in EVs, which spread in the whole brain to stimulate the aggregation of Aβ peptides and trigger neurotoxicity in neighboring neurons, and cause glial apoptosis 6, 8-10. EVs secreted from active astrocytes in the brain of patients with Parkinson's disease (PD) enhance the degeneration of dopaminergic neurons by enrichment of miR-34a 11 and aggravate motor neuron degeneration in the amyotrophic lateral sclerosis (ALS) model *via* transfer of depleted miR-494-3p 12, toxic SOD1, TDP-43, and FUS 6. Toxic EVs generated from active astrocytes are detrimental to synapse formation, axonal growth, and neuronal survival.

Recently, EVs have attracted wide attention due to their great therapeutic potential for various central nervous system diseases. EVs secreted from human bone marrow-derived mesenchymal stem cells have been reported to contribute to neurogenesis, reduce inflammation, and improve learning and memory functions 13. Exosomes derived from astrocytes in the traumatic brain restrain microglia-mediated neuroinflammation and ameliorate neurological deficits *via* miR-873a-5p by inhibiting the NF-κB signaling pathway 14. Exosomal cirSHOC2 derived from ischemic-preconditioned astrocytes suppresses autophagy via the miR-7670-3p/SIRT1 axis, thereby diminishing neuronal apoptosis 15. Also, EVs from astrocytes stimulated by ultrasound can reduce the Aβ plaque burden in APP/PS1 mice and protect neurons from damage 16.

Acidic fibroblast growth factor (aFGF) is a neurotrophic factor with neuroprotective functions involved in neurogenesis, synaptic plasticity regulation, and neuronal apoptosis inhibition 17-20. The level of aFGF is increased in the serum and cerebrospinal fluid of patients with AD 21. Our previous studies have demonstrated that Tat-haFGF_14-154_, human aFGF fused with Tat-PTD, reduces Aβ deposition and attenuates behavioral deficits in APP/PS1 mice 22, 23. Since excessive or persistent reactive astrocytes lead to chronic inflammation and neuronal dysfunction, and the activation of astrocytes can be inhibited by aFGF 24, we hypothesized that AEV cargoes might be modified by aFGF stimulation and play a role in AD progression. To verify this hypothesis, we isolated EVs from damaged astrocytes and treated them with or without aFGF under Aβ-loading conditions defined as AEVs_-Aβ+H_ and AEVs_-Aβ_, respectively. We measured the ability of AEVs to enter the brain, ameliorate cognitive behavior deficits, alleviate Aβ burden, and improve synapse ultrastructure. Subsequently, the miRNAs enriched in AEVs were sequenced to identify the key molecules specifically modified by aFGF. Finally, we explored the protective effects of miR-206-3p inhibition on cognitive deficiency and its regulatory mechanism and determined the role of miR-206-3p as a specific biomarker for potential AD diagnosis.

## Materials and Methods

### Isolation and culture of rat cortical neurons and astrocytes

Primary cortical neurons and astrocytes were isolated from the brain of neonatal rats (Sprague-Dawley) according to the methods described previously with minor modifications 23, 25. The brain tissue was minced and digested with 0.125% trypsin at 37 °C. Subsequently, the suspension was passed through a 200-mesh filter. For primary cortical neurons, after culturing for 6 h, Dulbecco's Modified Eagle's Medium (DMEM) was replaced with the neurobasal medium (Invitrogen, USA) containing 2% B27 supplement (50×, life technology, USA). Primary cortical neurons were used in experiments on day 5. For primary astrocytes, the supernatant was transferred to a polylysine-coated 25-cm^2^ flask and cultured for 8 days. When the cells reached 90% confluency, microglia and precursor cells were removed by shaking. The astrocyte biomarker, glial fibrillary acidic protein (GFAP), was used to determine the purity of isolated astrocytes.

### Isolation and identification of AEVs

Astrocytes derived from neonatal rats were divided into three groups. For the Aβ_1-42_ injury group, astrocytes were incubated with DMEM-F12 + 2% exosome-free fetal bovine serum (FBS) (EXO-FBS-50A-1, SBI, USA), and 4 μM Aβ_1-42_ for 72 h. For the Aβ_1-42_ and haFGF_14-154_ co-treatment group, astrocytes were incubated with DMEM-F12 medium + 2% exosome-free FBS, 4 μM Aβ_1-42,_ and 1000 ng/mL haFGF_14-154_ (Aβ+H) for 72 h. The volume-matched vehicle was added to the control group. The collected supernatant was centrifuged and passed through 0.22 μm filters (Millipore, Billerica, MA, USA) to remove cell fragments. AEVs (AEVs-_Ctrl_, AEVs-_Aβ_ and AEVs-_Aβ+H_) were collected from the supernatant of astrocytes subjected to different treatments using the total exosome isolation reagent (Invitrogen by Life Technologies, USA, 4478359) according to the manufacturer's instructions. For AEVs identification, EV biomarkers CD9 (1:1000, Rabbit, Affinity), CD63 (1:1000, Rabbit, Affinity), and clathrin (1:2000, Mouse, BD, 610499) were detected by immunoblotting, and endoplasmic reticulum-specific biomarker calnexin (1:1000, Rabbit, Absin, abs131462) served as a negative biomarker. The particle size and number of AEVs were detected and analyzed using a Multiple-Laser Zeta View f-NTA Nanoparticle Tracking Analyzer (Particle Metrix, Meerbusch, Germany). Diluted AEVs in deionized water were spotted onto the cleaved mica sheets and dispersed evenly, and the samples were observed and imaged using an atomic force microscope (AFM) (Dimension Icon; Bruker Corp, Billerica, MA, USA).

Rat primary cortical neurons (5 DIV) were treated with 4 μM Aβ_1-42_ oligomer and 20 μg AEVs (AEVs-_Ctrl_, AEVs-_Aβ_, and AEVs-_Aβ+H_) for 24 h. Aβ_1-42_ powder (Qiangyao Biotechnology, Shanghai, China) was dissolved in 0.1% dimethyl sulfoxide (DMSO), disrupted by ultrasonic treatment on ice, and incubated at 37 °C for 24 h. The uptake of AEVs was tracked by incubating neurons with 5 μg AEVs labeled with red-fluorescent lipophilic dye PKH26 (Sigma-Aldrich, MIDI26, USA) and 4 μM Aβ_1-42_ oligomer for 0.5 h. Subsequently, neurons stained as MAP2^+^ cells (green) and labeled AEVs (red) were visualized under confocal microscopy (LSM 710 META, Carl Zeiss, Germany).

### miRNA microarray analysis of AEVs

Total RNA of AEVs was extracted and quantified by NanoDrop ND-2000 (ThermoFisher Scientific, USA). Small RNAs were ligated to RNA 3'- and 5'-adapter and transcribed into first-strand cDNA, followed by PCR amplification. Subsequently, the target fragment library was collected by gel extraction. After quality determination, the target fragment library was sequenced using the Illumina HiSeq^TM^ 2500 system. sRNA deep-sequencing and data analysis were conducted by Ribobio company (Guangzhou, China).

### Treatment of animals

Two-month-old male C57BL/6J (24-28 g) mice were purchased from the animal research center of Zhejiang ViTal River Technology Co. Seven-month-old male APPswe/PS1d9 double transgenic mice (APP/PS1) and wild-type (wt) male mice (APP/PS1-wt) were obtained from Cavens Biogle Model Animal Research Co. (Jiangsu, China). All animals were acclimatized for 14 days under controlled conditions of temperature (24 ± 1 °C) and humidity (50%-60%) with a 12 h-light/dark cycle before treatment. All animals were housed in the specific-pathogen-free environment of the laboratory experiment research center (Jinan University), and experiments were performed following the Chinese Institutional Guidelines for Animal Care and Use and approved by the animal ethics committee of Jinan University (approval number: 20201028-07). AD was induced by slowly injecting 4 μL of Aβ_1-42_ oligomer solution (2 μg/μL, 4 μg/side and 8 μg/mouse) into the bilateral hippocampus (bregma, ± 1.5 mm lateral, -2.3 mm posterior, 2.36 mm deep) using a micro-injection pump at the rate of 0.8 μL/min. After three weeks of the Aβ brain injection, each mouse was injected with 2.5 nmol miR-206-3p antagomir or miRNA antagomir NC (RiboBio, Guangzhou, China) dissolved in 4 μL phosphate-buffered saline (PBS) into the bilateral hippocampus (bregma, ± 1.5 mm lateral, -2.3 mm posterior, 2.36 mm deep) using a micro-injection pump at the rate of 0.8 μL/min. All mice in the sham group or Aβ group were injected with an equal volume of PBS.

AEVs and volume-matched PBS were intranasally administered to APP/PS1 mice and AD model mice induced by brain injection of Aβ_1-42_ oligomer every 2 days for 6 weeks according to the previous protocol 23. AEVs (AEVs-_Ctrl_, AEVs-_Aβ_ and AEVs-_Aβ+H_) were resuspended in sterile PBS at the final concentration of 4 μg/μL. Each nostril was treated with 3 μL hyaluronidase (100 U/mouse, S10060, Shanghai Yuanye Bio-Technology Co., Ltd) for 30 min 13 to permeabilize the nasal mucous membrane. Subsequently, each mouse was gently held with the ventral side up, and the head was facing downward. Each nostril received a total volume of ~8 μL of AEVs or PBS (~2.5 μL each time, lasted for 3 min) by a 10-μL micropipette. For tracking AEVs in the brain, 60 μg of AEVs labeled with PKH26 or an equal volume of PBS were intranasally administered to APP/PS1 mice. After 6 h, mice were sacrificed, and the distribution of AEVs in the olfactory bulb (OB) and entorhinal cortex was observed by confocal microscopy.

### Y-maze and Morris water maze

The Y-maze test was conducted to investigate short-term memory as described previously 26. The device (30 cm × 8 cm × 15 cm) consisted of three arms labeled as A, B, and C at a 120° angle from each other. Each mouse was placed in the A-arm facing the wall to freely explore the maze for 8 min. The movement tracks, total arm entries, percentage of alternation triples, and total distance were recorded using a camera controlled by the super-maze software (Shanghai Xin Ruan Information Technology Co.). The percentage of spontaneous alternation for each mouse was calculated by the following formula: spontaneous alternation rate (%) = (number of successful alternations/number of total arms entries-2) ×100%. Pearson's correlation of spontaneous alternation to total arm entries and the total distance of movement was analyzed to exclude experimental interference factors. The Morris water maze (MWM) test was conducted 72 hours after finishing Y-maze according to the previous protocol 27. The MWM device consisted of a circular pool (120 cm diameter, 50 cm height) surrounded by visible black cues and a submersible platform (8 cm diameter) for animals to escape. The camera was used to record the movement tracks in real-time by the super-maze software (Shanghai Xin Ruan Information Technology Co.) installed on the computer. At the start of the navigation test, a hidden platform (1 cm below the water level) was kept in the middle of one quadrant throughout the training phase. Each mouse was trained for 6 days with one trial per quadrant and two different quadrants per day (interval between each trial over 20 min). The quadrants acted as entry to the pool, excluding the target and opposite quadrant. The escaped latency time and movement tracks of each mouse were recorded in 60 s. If the mouse found and stayed on the platform for 4 s, the system automatically stopped recording. On the 6th day, the platform under the water was removed, and the spatial probe test was performed. The times of crossing the removed-platform area and time spent in the target quadrant of the platform were recorded within 90 s.

### Cellular immunofluorescence and tissue immunohistochemistry

The cells fixed with 4% paraformaldehyde and frozen tissue specimens were permeabilized with 0.2% triton-X-100 (Sigma) for 20 min and blocked in 5% bovine serum albumin (BSA) for 1 h at room temperature. Then the samples were incubated overnight at 4 °C with 1:200 diluted mouse monoclonal anti-MAP_2_ antibody (Abcam, ab11267) and 1:300 diluted rabbit monoclonal anti-GFAP antibody (CST, D1F4Q). The next day, the samples were incubated with diluted Alexa flour^®^ 594 goat anti-mouse IgG (Life Technologies, 1:300, A11005) or Alexa flour^®^ 488 goat anti-rabbit IgG (Life Technologies, 1:300, A11008) for 1 h at room temperature. Subsequently, nuclei were stained with DAPI for 10 min. Images were captured with a confocal microscope (LSM 710 META, Carl Zeiss, Germany) and a light microscope (Nikon, Japan), and the results were analyzed by image-pro-plus software.

The frozen hippocampal tissue specimens were thawed at room temperature for 20 min and treated with 3% H_2_O_2_ solution for 10 min to remove endogenous enzymes. The specimens were boiled in 0.01 M citrate buffer solution (pH 6.0) for 15 min to repair the antigen, incubated with 70% formic acid for 30 min, and blocked by 5% BSA for 1 h at room temperature. Next, specimens were incubated with mouse anti-6E10 antibody (1:200, Biolegend, 803014) overnight at 4 °C. On day 2, the specimens were incubated with anti-mouse IgG-horseradish peroxidase (HRP, Boster Biotech, SV0001) secondary antibody for 1 h at 37 °C, followed by staining with diaminobenzidine (DAB, Servicebio, G1212) and hematoxylin (Boster Biotech, AR0005) for 1-2 min. The images of slides were obtained by light microscope and analyzed by Image-Pro Plus software.

### Western blotting

Tissue or cell samples were lysed on ice for 30 min in the RIPA buffer containing PMSF and phosphatase inhibitors and the lysates were centrifugated at 1,2000 rpm for 30 min at 4 °C. The protein concentration of supernatants was quantified by using the bicinchoninic acid (BCA) protein assay kit (ThermoFisher Scientific). After 12% SDS-PAGE gel separation, proteins were transferred to polyvinylidene difluoride (PVDF) membranes (Millipore, Billerica, USA), and the membranes were blocked by 5% fat-free milk for 1 h at room temperature followed by incubation with the primary antibody overnight at 4 °C. The primary antibodies included rabbit monoclonal anti-PSD95 (1:1000, CST, D74D3), rabbit monoclonal anti-GAP43 (1:1000, Abcam, ab75810), rabbit monoclonal anti-synaptophysin (SYN) (1:1000, Abcam, ab32127), rabbit monoclonal anti-brain-derived neurotrophic factor (BDNF) (1:1000, Abcam, ab108319), mouse monoclonal anti-6E10 (1:1000, Biolegend, 803014), rabbit monoclonal anti-AEP (1:1000, CST, 93627S), mouse monoclonal anti-β-actin (1:5000, Bioworld, BS6007M), and mouse monoclonal anti-β-III-tubulin (1:5000, Abcam, ab52623) antibodies. The membranes were incubated with the secondary antibody (peroxidase-conjugated affinity-purified goat anti-rabbit/mouse IgG, 1:5000, FD Biotechnology, China) for 1 h at room temperature, and blots were detected by ECL kit (Thermo Fisher Scientific, USA) under the imaging system (Tanon, Shanghai, China).

### Enzyme-linked immunosorbent assay (ELISA)

Mouse cortical and hippocampal lysates were added to a monoclonal BDNF antibody pre-coated 96-well plate following the instructions of ELISA Kit (Multiscience, EK2127-96), and the absorbance of the samples was read at 450 nm and 570 nm wavelengths using the Multiskan GO Spectrophotometer (Thermo Scientific). The BDNF concentration in the cortex and hippocampus was calculated using the standard reference samples and further calibrated with protein concentration in tissue lysates.

### Structure of hippocampal synapses by transmission electron microscopy (TEM)

The hippocampal CA1 region of APP/PS1 mice was fixed with 2.5% glutaraldehyde (Sigma-Aldrich) overnight at 4 °C. The specimens were placed in 1% oxalic acid at 4 °C for 1.5 h followed by dehydration in a series of graded ethanol solutions (70%, 80%, 90%, 95%, and 100% for 10 min). Subsequently, the samples were permeated with acetone embedding agent mixture (1:1) for 1 h and (1:3) overnight. After drying ultrathin sections on the copper mesh, the tissue structure was observed using TEM (TECNAI G2 Spirit TWIN, FEI), and the length and width of postsynaptic density (PSD) were analyzed by image-pro plus software.

### RNA isolation and RT-qPCR assay

miRNAs were isolated using the Trizol reagent (Invitrogen), and first chain cDNA was synthesized with the All-in-one miRNA First-Strand cDNA synthesis kit (GeneCopoeia, QP013) following the manufacturer's instructions. cDNAs were amplified as per All-in-One miRNA qRT-PCR Detection Kit (GeneCopoeia, QP016) instructions, and the fluorescence signal was captured by a Bio-rad PCR system. The U6 small RNA (GeneCopoeia) or cel-miR-39-3p (GeneCopoeia) was used to normalize miRNAs. The primers (rno-miR-130b-5p and rno-miR-224-5p Art.No. miRA10000) were designed and synthesized by Ribobio (Guangzhou, China). The primers (rno-miR-206-3p: Art.No. RmiRQP0308; rno-miR-196-5p: Art.No. RmiRQP0284; rno-miR-18a-5p: Art.No. RmiRQP0255; rno-miR-344b-1-3p: Art.No. RmiRQP1887; rno-miR-92a-1-5p: Art.No. RmiRQP3100; rno-miR-485-3p: Art.No. RmiRQ3252; rno-miR-204-3p: Art.No. RmiRQP1203; rno-miR-133a-p: Art.No. RmiRQP0166; mmu-miR-206-3p: Art.No. MmiRQP0308; RSnRNAU6: Art.No. RmiRQP9003; MSnRNAU6: Art.No. MmiRQP9002) were designed and synthesized by GeneCopoeia (Guangzhou, China).

### *In situ* hybridization of miR-206-3p

After 4% paraformaldehyde fixation, the right hemispheres of APP/PS1 mice were embedded with paraffin and cut into 6-μm thick sections. The sections were dewaxed and treated with 0.2 M HCl and 0.5% triton-X-100 for 12 min. Next, the specimens were treated with 20 μg/mL protease K (Focobio) for 12 min at room temperature and incubated with 1:100 diluted probes (Focobio) for 7 min at 80 °C and 10 min at 37 °C. After hybridization at 41 °C for 48 h, the specimens were processed using the following steps: washing with PBS, incubating with 1:100 diluted anti-digoxin HRP (Focobio) for 1 h, and subsequently with TSA (green, Focobio) for 15 min at 37 °C. After nuclear staining, sample images were observed by a confocal microscope.

### Transfection of neurons with miR-206-3p mimic and inhibitor

Primary cortical neurons (5 DIV) were transfected with 40 nM miR-206-3p mimics (the endogenous miR-206-3p mimic was synthesized by chemical synthesis) or miR-206-3p inhibitors and the corresponding negative control (NC) for 48 h. miR-206-3p mimics (sense sequences 5'-3': UGGAAUGUAAGGAAGUGUGUGG, antisense 5'-3': ACACACUUCCUUACAUUCCAUU), mimic negative control (mimic NC, sense 5'-3': UUCUCCGAACGUGUCACGUTT; antisense 5'-3': ACGUGACACGUUCGGAGAATT), miR-206-3p inhibitors (sequences 5'-3': CCACACACUUCCUUACAUUCCA), inhibitor negative control (inhibitor NC, sequences 5'-3': CAGUACUUUUGUGUAGUACAA) were designed and synthesized by Genpharma (Shanghai, China). The protein levels of BDNF, postsynaptic dense protein 95 (PSD95), growth-associated protein 43 (GAP43), and Synaptophysin (SYN) were measured by Western blotting, and neuronal morphology was observed by immunofluorescence with MAP2^+^ staining.

### Luciferase reporter assay

We designed wild-type and mutant BDNF 3'UTR sequences containing the predicted miR-206-3p binding sites (Sangon Biotechnology, Shanghai, China). The target fragments were inserted into the psiCHECK-2 vector to generate the wt-3'UTR-psiCHECK2 and mut-3'UTR-psiCHECK2 vectors. Subsequently, wt-3'UTR-psiCHECK2 or mut-3'UTR-psiCHECK2 plasmids with miR-206-3p mimics (Genpharma, Shanghai, China) were co-transfected into HEK293T cells using Lipofectamine 3000 (Invitrogen, USA). The relative luciferase activity was measured by the fluorescence intensity ratio of Renilla luciferase fluorescence and firefly luciferase fluorescence (internal control) according to the manufacturer's instructions of the dual-luciferase reporter system (Promega, USA). To exclude the target relationship between miR-206-3p and synaptic protein markers (PSD95, GAP43, and SYN), 3'UTR sequences of PSD95, GAP43, and SYN were synthesized by Genscript (Nanjing, China) and cloned into psiCHECK-2 vectors. Then, wt-PSD95-3'UTR-psiCHECK2, wt-GAP43-3'UTR-psiCHECK2, and wt-SYN-3'UTR-psiCHECK2 combined with miR-206-3p mimics were co-transfected in HEK293T cells. The dual-luciferase reporter assays were performed 48 h after transfection.

### Human plasma samples

A total of 70 plasma specimens from participants (patients with AD: n = 18; age-matched healthy control: n = 25; Parkinson's disease: n = 15; Schizophrenia: n = 12) were obtained from the Third Affiliated Hospital of Sun Yat-sen University (Guangzhou, China). The study was approved by the Ethics Committee of the First Affiliated Hospital of Jinan University (approval number: KYK-2021-028). The characteristics of participants are displayed in Table 1. BDNF levels in human plasma were measured using the human BDNF ELISA kit (Cusabio, CSB-E04501h) following the manufacturer's protocol. The miR-206-3p levels in human plasma were detected by RT-qPCR as described before.

### Statistical analysis

Graphpad Prism 6 (Graphpad Software, Inc) was used for statistical analysis, and data were presented as mean ± SEM. For the two-group comparison, data were analyzed by Student's *t-test*. For more than three groups, data were analyzed by one-way analysis of variance (one-way ANOVA) followed by Tukey's post-hoc multiple comparison or two-way analysis of variance (two-way ANOVA) with Tukey's post-hoc multiple comparison. The association between the two variables was evaluated by the Pearson correlation coefficient. For human plasma sample analysis, age was included as a covariate, and the principal component analysis (PCA) was conducted to estimate the comprehensive score of all samples. The analysis of covariance (ANCOVA) (adjusting for age) with post hoc Tukey significance was performed to determine between-group differences. *P* < 0.05 was regarded as significantly different.

## Results

### AEVs-_Aβ+H_ promote neurite outgrowth and diminish Aβ loading in neurons *in vitro*

Immunofluorescence staining showed that about 98% of astrocytes (3rd passage) derived from neonatal rats were positive for GFAP (Figure S1A). The rat cortical astrocytes were damaged by the Aβ_1-42_ oligomer (4 μM) used to simulate the microenvironment of the AD brain. haFGF_14-154_ (1 μg/mL) was added to the culture medium, and AEVs were isolated from the conditioned medium with or without Aβ_1-42_/haFGF_14-154_ treatment defined as AEVs-_Ctrl_, AEVs-_Aβ_, and AEVs-_Aβ+H_ (Figure S1B). The isolated AEVs were characterized by Western blotting, NTA, and AFM. The exosomal biomarkers, including CD9, CD63, and clathrin, were enriched in AEV lysates. The immunoblotting of calnexin was negative, indicating that purified AEVs were free of cellular debris (Figure S1C-E). CD63 and clathrin levels were significantly decreased in AEVs-_Aβ_ compared with AEVs-_Ctrl_, while there was no significant difference between AEVs-_Aβ+H_ and AEVs-_Ctrl_. Moreover, Aβ damage did not change levels of exosome biomarker proteins in astrocytes, and AEV secretion was inhibited in astrocytes subjected to Aβ concentration. Aβ_1-42_ has been reported to reduce exosome release in cultured astrocytes by stimulating the JNK signaling pathway 28. The size distributions determined by NTA showed a major peak at 115 nm in all AEVs with different treatments (Figure S1F), and the particles were spherical in shape as observed by AFM (Figure S1G). These data suggested that the main component of the harvested AEVs were exosomes.

**Figure 1:**
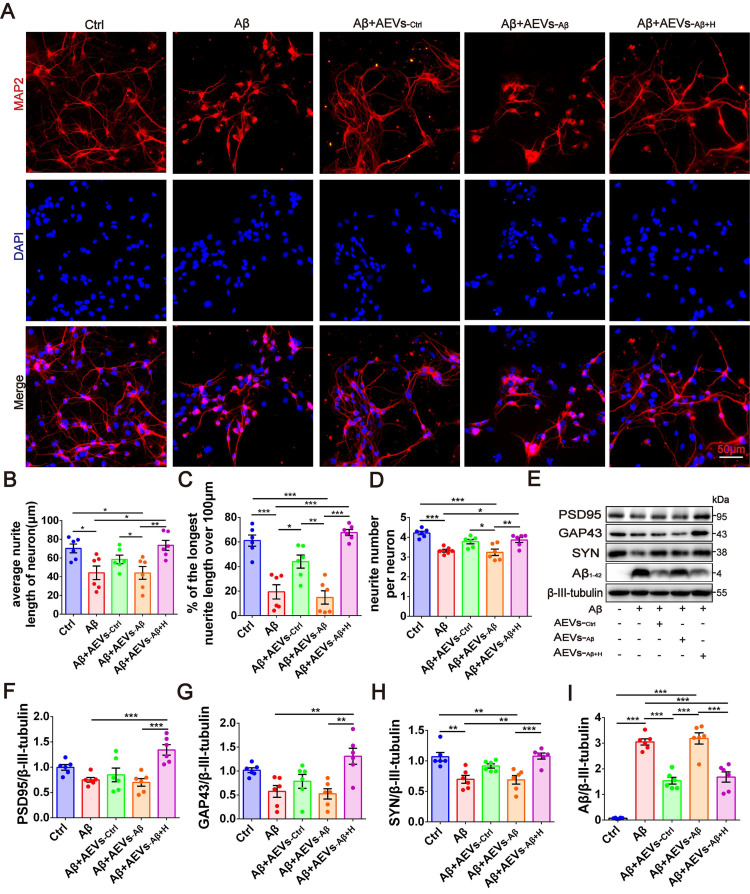
** AEVs-_Aβ+H_ promote neurite outgrowth and diminish Aβ loading in neurons *in vitro*.** (**A**) The representative images of MAP2^+^ cells (red) in rat primary cortical neurons treated with AEVs under the condition of Aβ-injury. (Scale bars, 50 µm). Quantification of the average neurite length of neurons (**B**), the proportion of the longest length of neural neurites over 100 µm (**C**) and the number of neural neurites per neuron (**D**), n = 6. (**E**) The representative immunoblots of PSD95, GAP43, SYN and Aβ. The quantified expression of PSD95 (**F**), GAP43 (**G**), SYN (**H**) and Aβ (**I**), n = 6. Data are mean ± SEM. *p < 0.05, **p < 0.01, ***p < 0.001. *P* values are calculated by one-way ANOVA with Tukey's post-hoc test.

Next, we investigated whether the Aβ_1-42_ oligomer had a protective effect on the damaged neurons in AEVs. The AEVs labeled with PKH26 (red) were added to the culture medium of primary cortical neurons (DIV 5). Following 30 min of incubation, AEVs were taken up by neurons and distributed into the cytoplasm (Figure S2A). After staining MAP2^+^ cells, neuronal morphology analysis showed that Aβ_1-42_ reduced the average neurite length, percentage of the longest neurite length (> 100 μm), and neurite number; AEVs-_Aβ+H_ significantly reversed Aβ_1-42_-induced damage in neurons with improvement of the three parameters related to neurite outgrowth, while AEVs-_Ctrl_ just increased the percentage of longest neurite length (Figure 1A-D). Aβ_1-42_ inhibited the expression level of SYN, but there were no significant changes in PSD95 and GAP43 levels. Compared with Aβ_1-42_ injury and AEVs-_Aβ_ treatment, AEVs-_Aβ+H_ enhanced PSD95, GAP43, and SYN expression levels in neurons (Figure 1E-H). We also determined the level of Aβ loading in primary cortical neurons and unexpectedly found that Aβ loading in neurons was notably decreased by AEVs-_Ctrl_ and AEVs-_Aβ+H_ (Figure 1E and 1I). These results suggested that AEVs stimulated by aFGF in AD pathological situations have favorable neuroprotection by enhancing neurite growth and reducing Aβ loading in neurons.

### AEVs-_Aβ+H_ ameliorate cognitive behavior deficits, promote synaptic plasticity, and alleviate brain Aβ burden in AD model mice

**Figure 2:**
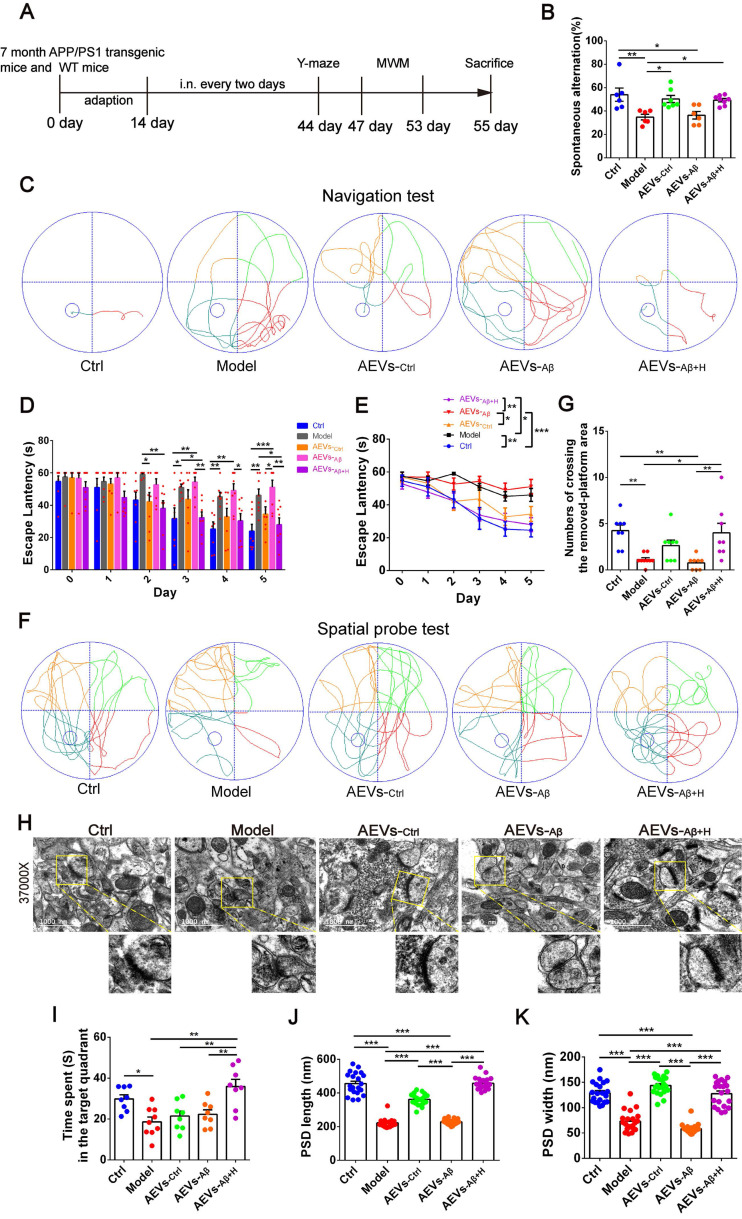
** AEVs-_Aβ+H_ ameliorate cognitive behavior deficits and promote synaptic plasticity in APP/PS1 mice.** (**A**) Schedule of intranasal administration and behavior test of APP/PS1 mice. (**B**) Spontaneous alternation in Y-maze (n = 6-8). (**C**) The representative movement tracks of escape latency on day 5 in navigation test of MWM. (**D-E**) The escape latency (n = 8/9). Representative movement tracks in spatial probe test (**F**), numbers of crossing the removed platform area (**G**) and time spend in target quadrant (**I**) in spatial probe test of MWM, n = 8/9. (**H**) The representative images of synaptic structure in CA1 region of hippocampus observed by TEM. The length (**J**) and width (**K**) of PSD were quantified from 7 random fields per slide, 1 slide per animal, and 3 animals per group (n = 21). Data are mean ± SEM. *p < 0.05, **p < 0.01, ***p < 0.001. *P* values are calculated by one-way ANOVA with Tukey's post-hoc test and two-way ANOVA with Tukey's post-hoc test.

AEVs were delivered into the brain of APP/PS1 mice by intranasal administration. It has been reported that EVs can enter the brain efficiently after intranasal administration 29. Consistent with this report, we found that the PKH26-labeled AEVs distributed in the olfactory bulb and entorhinal cortex 6 h after the intranasal administration (Figure S2B-C). AEVs were intranasally administered to 7-month-old APP/PS1 mice every 2 days for 6 weeks, followed by Y-maze and MWM tests (Figure 2A). As shown in Figure 2B, AEVs-_Ctrl_ and AEVs-_Aβ+H_ attenuated short-term memory deficits in APP/PS1 mice, characterized by increased spontaneous alternation in the Y-maze test. No significant differences were observed in total arm entries and total traveled distance in mice with different treatments (Figure S3A-D), indicating that the improvement in spontaneous alternation could not be attributed to locomotor activity. Two days after finishing the Y-maze test, MWM was performed to estimate the spatial learning and memory abilities in APP/PS1 mice treated with AEVs. The representative movement tracks of escape latency on day 5 are displayed in Figure 2C. The mice with intranasal administration of AEVs-_Ctrl_ and AEVs-_Aβ+H_ demonstrated a significant reduction in escape latency (Figure 2D-E) and path length (Figure S3E) compared with non-treated or AEVs-_Aβ_-treated APP/PS1 mice. In the spatial probe test, APP/PS1 mice treated with AEVs-_Aβ+H_ displayed improved performance after the training, as evidenced by the increased numbers of crossing over the removed-platform area and time spent in the target quadrant (Figure 2F-G and 2I).

**Figure 3:**
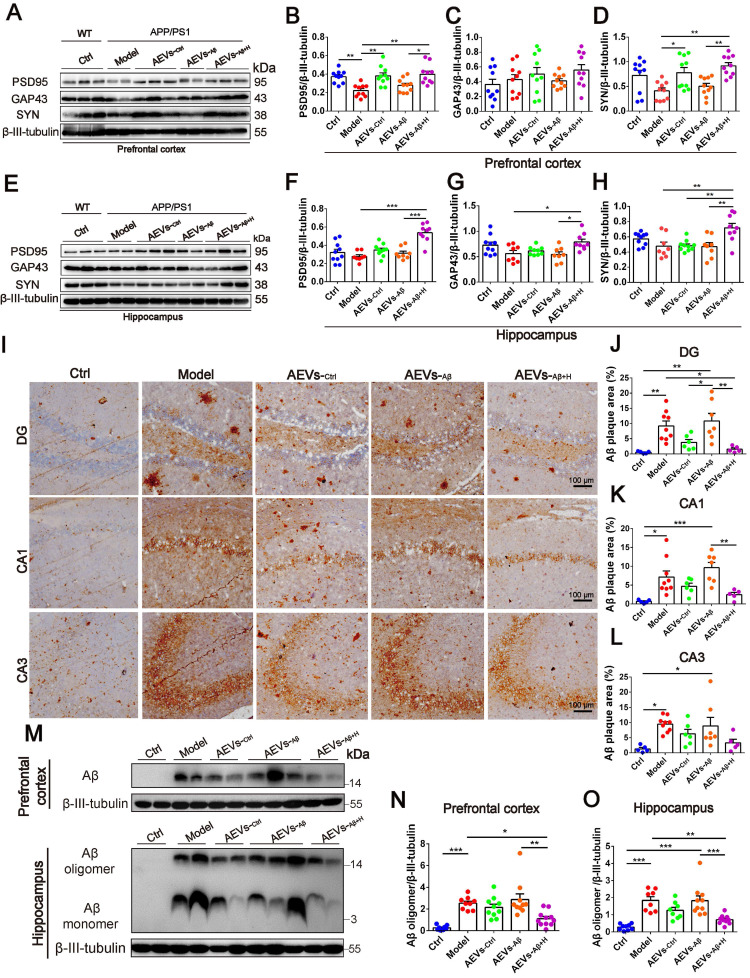
** AEVs-_Aβ+H_ enhance the expression of synaptic proteins and alleviate brain Aβ burden in APP/PS1 mice.** (**A-H**) The expression of PSD95, GAP43 and SYN in the prefrontal cortex (PFC) (**A**) and hippocampus (**E**) of APP/PS1 mice with intranasal administration of AEVs. The relative expression quantification of PSD95 (**B**), GAP43 (**C**), SYN (**D**) in the PFC and PSD95 (**F**), GAP43 (**G**), SYN (**H**) in hippocampus, n = 8-10. (**I**) The representative images of Aβ plaque in the dentate gyrus (DG), CA1 and CA3 regions of hippocampus (Scale: 100 µm). (**J-L**) The area of Aβ plaque is analyzed by image-pro plus software, n = 5-7. (**M**) Aβ in the PFC and hippocampus were detected by western blot. The quantified expression of Aβ in the PFC (**N**) and hippocampus (**O**), n = 8-10. Data are mean ± SEM. *p < 0.05, **p < 0.01, ***p < 0.001. *P* values are calculated by one-way ANOVA with Tukey's post-hoc test.

The hippocampal CA1 region is considered the major area involved in learning and memory in the brain. Therefore, we examined the synaptic ultrastructure of the CA1 region in APP/PS1 mice treated with AEVs. The ultrastructural analysis showed that AEVs-_Aβ+H_ and AEVs-_Ctrl_ treatment significantly elevated the PSD length and width of synapses in the hippocampal CA1 region of APP/PS1 mice (Figure 2H and 2J-K). APP/PS1 mice showed decreased expression of SYN and PSD95 in the prefrontal cortex. AEVs-_Aβ+H_ and AEVs-_Ctrl_ enhanced the expression of SYN and PSD95, consistent with the synaptic stabilization improvement. Moreover, AEVs-_Aβ+H_ enhanced the expression of SYN, PSD95, and GAP43 in the hippocampus (Figure 3A-H).

Aβ plaque deposition is one of the most important pathological characteristics in APP/PS1 mice. We detected Aβ plaques by immunohistochemistry and Aβ oligomers by Western blotting in the prefrontal cortex and hippocampus. AEVs-_Aβ+H_ significantly reduced the Aβ burden in the hippocampus (DG region) of APP/PS1 mice, but AEVs-_Ctrl_ non-significantly reduced the Aβ burden (Figure 3I-L). Consistently, immunoblotting indicated that AEVs-_Aβ+H_ treatment significantly decreased the Aβ oligomer level in the prefrontal cortex and hippocampus of APP/PS1 mice, whereas a non-significant decrease was observed with the AEVs-_Ctrl_ treatment (Figure 3M-O).

To further confirm the improvement effects of AEVs-_Aβ+H_ on AD mice, we developed another AD mouse model by bilateral hippocampus injection of the Aβ_1-42_ oligomer, and AEVs were intranasally delivered to mice every 2 days for 6 weeks (Figure S4A). In the Y-maze test, compared with sham-operated mice, the percentage of spontaneous alternation significantly reduced in mice with brain injection of Aβ_1-42,_ but the reduction was reversed by AEVs-_Aβ+H_ (Figure S4B). There was no significant correlation between the percentage of spontaneous alternation and total arm entries or total distance traveled during the test (Figure S4C-F). The representative movement tracks of escape latency in the MWM test on day 5 are shown in Figure S4G. Consistent with the results of the Y maze, AD model mice spent a longer time arriving at the platform than the sham-operated mice, while AEVs-_Aβ+H_ treatment remarkably shortened the escape latency and increased the number of crossing over the removed-platform area and time spent in the target quadrant (Figure S4H-M). AD model mice treated with AEVs-_Ctrl_ did not display improved performance in the Y maze and MWM test, as evidenced by the insignificant change in the spontaneous alternation, escape latency, number of crossing over the removed-platform area and time spent in the target quadrant compared with the model mice. AEVs-_Aβ+H_ enhanced the level of PSD95 and GAP43 in the prefrontal cortex and upregulated the level of SYN in the hippocampus of AD mice (Figure S5A-H). Additionally, immunohistochemistry and immunoblotting results revealed that AEVs-_Aβ+H_ significantly reduced the Aβ burden in the prefrontal cortex and hippocampus (Figure S6).

These findings suggested that AEVs secreted in the AD brain (AEVs-_Aβ_) did not ameliorate Alzheimer's phenotype while AEVs modified by aFGF stimulation in Aβ-loading condition (AEVs-_Aβ+H_) attenuated the cognitive behavior deficits and Aβ burden accompanied with improved neurite growth and synaptic stabilization. AEVs-_Aβ+H_ had beneficial effects on AD similar to AEVs produced in a normal situation (AEVs-_Ctrl_).

### miR-206-3p level in AEVs is downregulated by aFGF in the AD microenvironment

**Figure 4:**
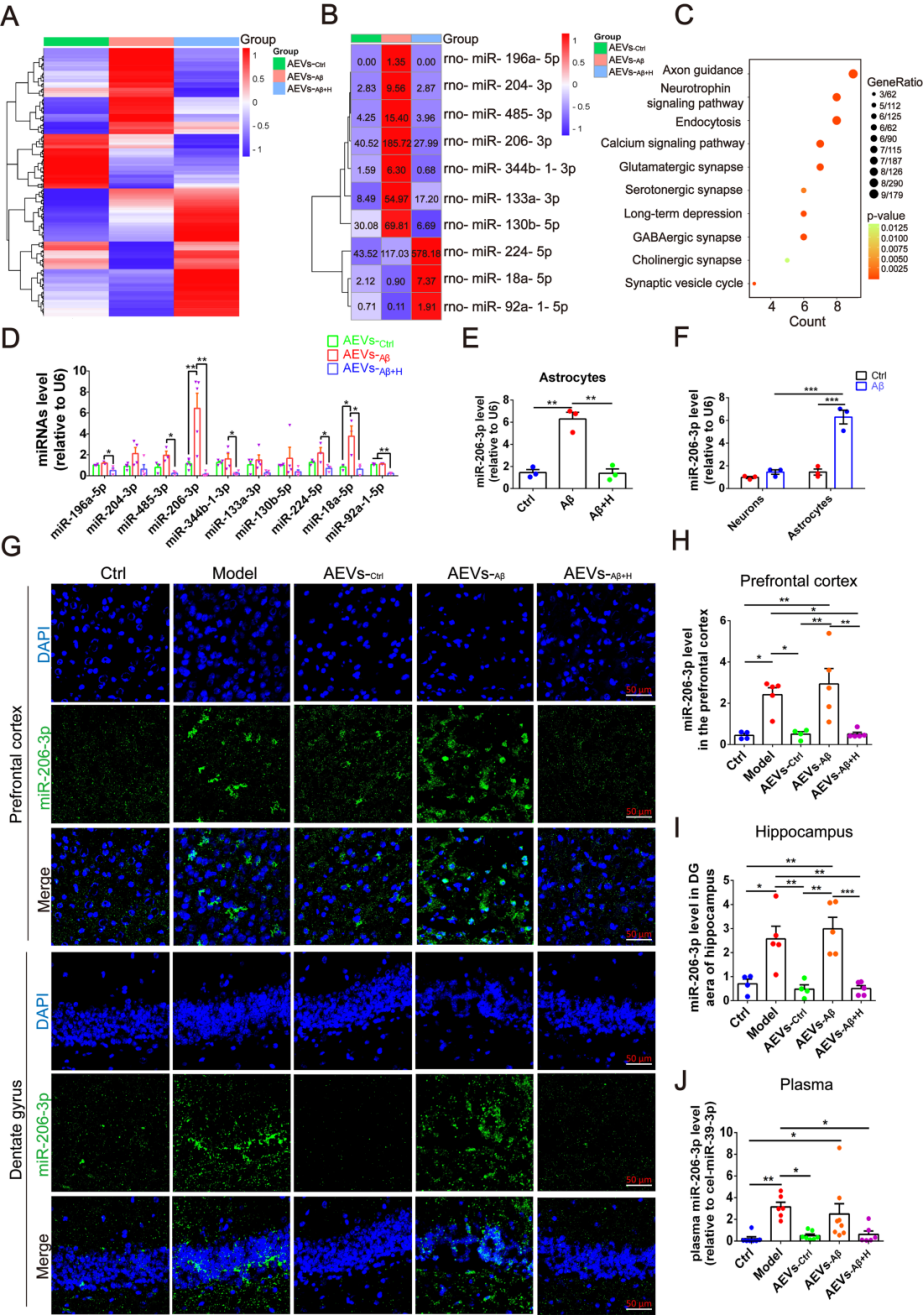
** aFGF down-regulates the level of miR-206-3p targeting BDNF in AEVs secreted in AD microenvironment.** (**A**) Heatmap of miRNA expression profile showing the expression difference in AEVs-_Ctrl_, AEVs-_Aβ_ and AEVs-_Aβ+H_ by color bar. Red represents increased expression and blue represents decreased expression. Scale is 1.0 to -1.0. (**B**) Heatmap image displays the significant difference of miRNAs in AEVs-_Ctrl_, AEVs-_Aβ_ and AEVs-_Aβ+H_, and the miRNA level is showed as number in each square. (**C**) Representative signaling pathways of genes targeted by significantly differential miRNAs according to KEGG analysis. (**D**) The differential miRNAs verified by qPCR in AEVs, n = 3-5. (**E**) The expression of miR-206-3p detected by qPCR in astrocytes damaged by Aβ accompanied with haFGF_14-154_ treatment, n = 3. (**F**) Comparison of miR-206-3p level between neurons and astrocytes subjected to Aβ_1-42_ oligomer, n = 3/4. (**G**) The distribution of miR-206-3p (green) in the PFC and hippocampal DG region of APP/PS1 mice measured by FISH and the quantified levels in the PFC (**H**) and DG region (**I**)**,** n = 4/5. (**J**) The plasma miR-206-3p level detected by qPCR in wild-type mice and APP/PS1 mice with intranasal administration of AEVs or PBS, n = 6-8. Data are mean ± SEM. *p < 0.05, **p < 0.01, ***p < 0.001. *P* values are calculated by one-way ANOVA with Tukey's post-hoc test and two-way ANOVA with Tukey's post-hoc test.

Based on the above results, we next sought to explore the underlying mechanism of AEVs-_Aβ+H_ in alleviating the learning and memory deficits in AD mice by identifying the miRNAs enriched in AEVs. The overall expression level of miRNAs was analyzed by the number of reads per million clean tags (RPM) density distribution map (Figure S7A), and significantly differentially expressed miRNAs are displayed in the volcano map (Figure S7B-D). We identified almost 400 miRNAs enriched in AEVs, of which 10 miRNAs had a differential level of more than 3-fold. Seven of 10 miRNAs were upregulated in AEVs-_Aβ_ compared to AEVs-_Ctrl_, but the level of these miRNAs was remarkably suppressed in AEVs-_Aβ+H_ (Figure 4A-B). KEGG pathway classification showed that the target genes of 10 significantly differential miRNAs are enriched in various pathways, including axonal guidance, neurotrophic signal pathway, and endocytosis (Figure 4C). The differentially expressed miRNAs in AEVs were confirmed by qPCR (Figure 4D). Interestingly, the level of change of miR-206-3p was the most significant among the differentially expressed miRNAs. The intracellular miR-206-3p level was increased approximately 4.34-fold in astrocytes damaged by Aβ but returned to the normal level by aFGF treatment (Figure 4E). The target genes of miR-206-3p are involved in several top-ranked pathways enriched by KEGG pathway classification, including axonal guidance, neurotrophic signal pathway, endocytosis, glutamatergic synapse, and long-term depression (Figure 4B), suggesting miR-206-3p as an important target of AEVs-_Aβ+H_ for alleviating neurodegeneration.

We compared the miR-206-3p level in Aβ-damaged neurons and astrocytes and found it to be approximately 4.47-fold higher in astrocytes than in neurons (Figure 4F), suggesting that miR-206-3p is mainly produced by astrocytes but not by neurons in the AD microenvironment. To investigate the level of miR-206-3p in the AD brain, we detected miR-206-3p expression in the brain and plasma by FISH and qPCR, respectively. High levels of miR-206-3p were observed in the prefrontal cortex and hippocampal DG region of APP/PS1 mice compared with the normal control mice. miR-206-3p expression was significantly reduced in the hippocampal DG region and plasma of APP/PS1 mice intranasally treated with AEVs-_Aβ+H_ and AEVs-_Ctrl_ (Figure 4G-I). Nogueras-Ortiz *et al.* 30 reported that AEVs and EVs derived from neurons (NEVs) could be isolated from the plasma of AD patients, suggesting that the high miR-206-3p level secreted from the brain can be detected in plasma. Interestingly, a high miR-206-3p level was observed in the plasma of APP/PS1 mice, while AEVs-_Aβ+H_ and AEVs-_Ctrl_ reduced the miR-206-3p level (Figure 4J).

### miR-206-3p antagomirs promote neurite growth and alleviate AD phenotype

**Figure 5:**
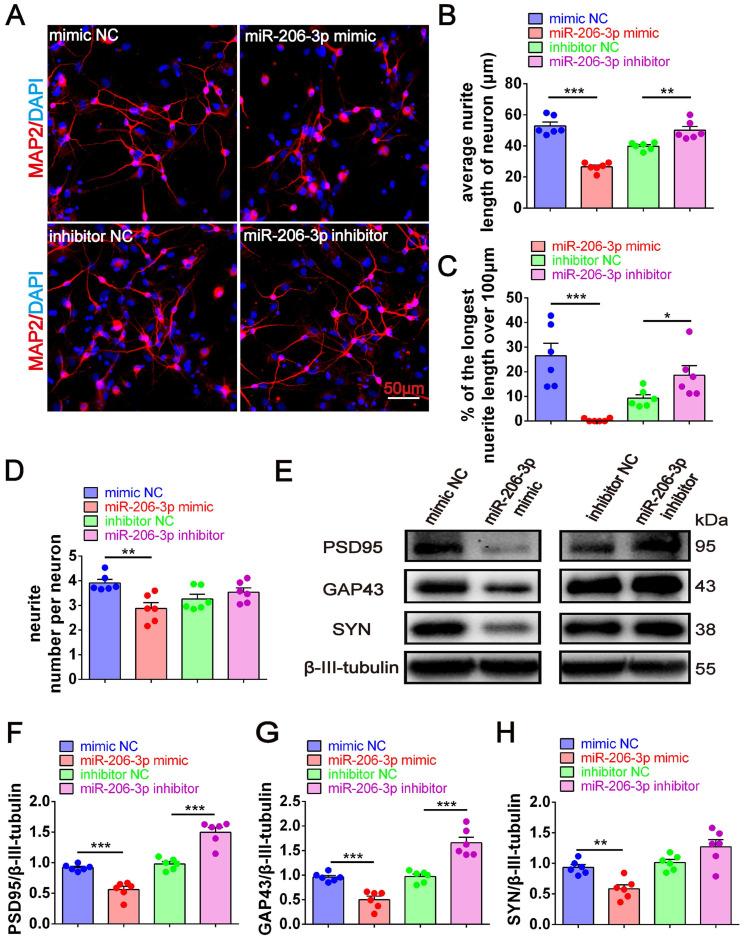
** miR-206-3p inhibitors promote neurite growth.** (**A**) Representative morphology of MAP2^+^ cells in primary cortical neurons (red) transfected with miR-206-3p mimics and inhibitors (Scale bars: 50 µm). Quantification of the average neurite length of neurons (**B**), the proportion of the longest length of neural neurites over 100 µm (**C**) and the number of neural neurites per neuron (**D**), n = 6. (**E-H**) The expression of PSD95, GAP43 and SYN in rat primary cortical neurons transfected with miR-206-3p mimics and inhibitors, n = 6. Data are mean ± SEM. *p < 0.05, **p < 0.01, ***p < 0.001. *P* values are calculated by student's *t-test*.

To further confirm the key role of depleted miR-206-3p in neuroprotection, we performed overexpression and knockdown of miR-206-3p in primary cortical neurons *in vitro*. miR-206-3p mimic-transfected neurons displayed a shorter average neurite length, a lower percentage of the longest neurite length (> 100 μm), and fewer average number of neurites than the negative control. However, miR-206-3p inhibitors increased the average neurite length and percentage of the longest neurite length (> 100 μm) in neurons (Figure 5A-5D). miR-206-3p mimics significantly inhibited the expression of PSD95, GAP43, and SYN compared with the corresponding controls, while the levels of PSD95 and GAP43 were remarkably enhanced by miR-206-3p inhibitors (Figure 5E-H).

**Figure 6:**
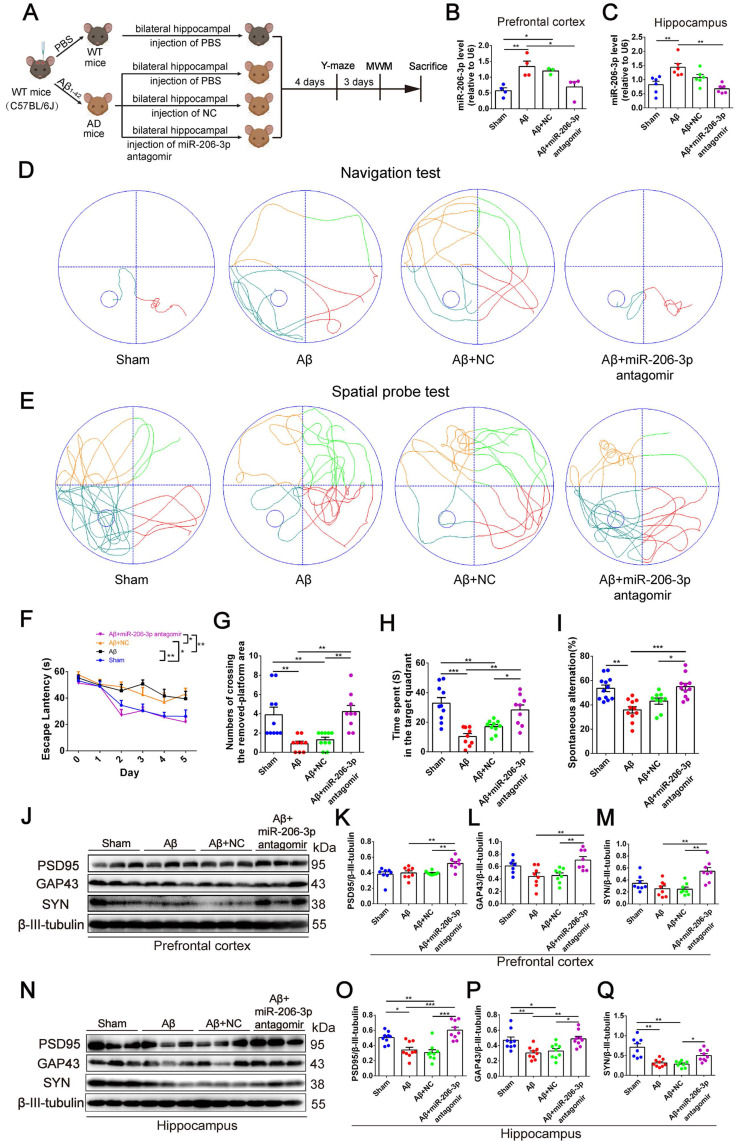
** miR-206-3p antagomirs alleviate learning and memory deficits in AD mice. (A)** Schematic protocol for the hippocampal injection of miR-206-3p antagomirs and behavior test. (**B-C**) miR-206-3p level in the PFC (**B,** n = 4) and hippocampus (**C**, n = 6) of AD mice with brain injection of miR-206-3p antagomirs. (**D**) The representative movement tracks of escape latency on day 5 in navigation test of MWM. (**F**) The escape latencies of mice. Representative movement tracks in spatial probe test (**E**), numbers of crossing the removed platform area (**G**) and time spend in target quadrant (**H**) in spatial probe test of MWM, n = 9/10. (**I**) Spontaneous alternation in Y-maze, n = 9-12. The expression of PSD95, GAP43 and SYN in the PFC (**J-M**) and hippocampus (**N-Q**) of AD mice with brain injection of miR-206-3p antagomirs and antagomir NC, n = 8/9. Data are mean ± SEM. *p < 0.05, **p < 0.01, ***p < 0.001. *P* values are calculated by one-way ANOVA with Tukey's post-hoc test and two-way ANOVA with Tukey's post-hoc test.

**Figure 7:**
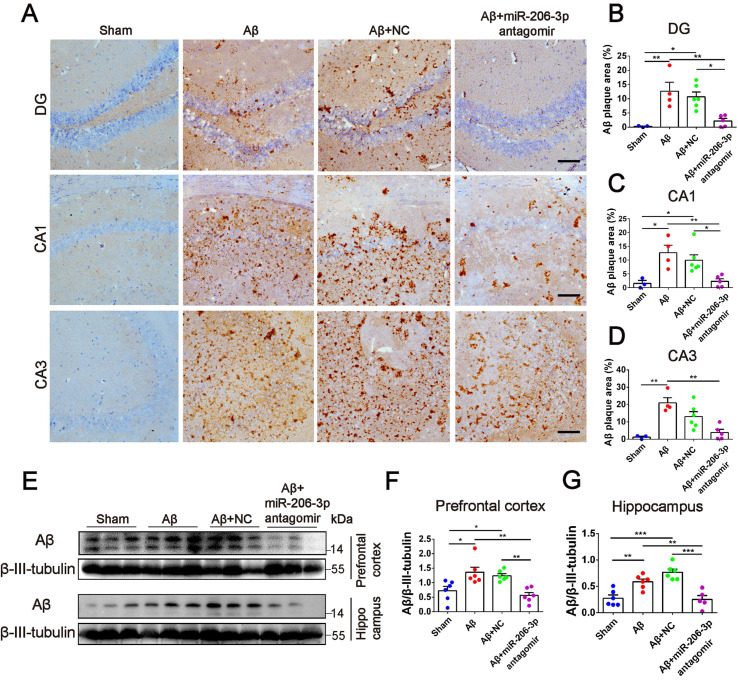
** miR-206-3p antagomirs reduce the deposition of Aβ in AD mice.** (**A**) Representative images of Aβ plaque in the DG, CA1 and CA3 regions of AD mice with hippocampal injection of miR-206-3p antagomirs (Scale: 100 µm). (**B-D**) Quantitative analysis for Aβ plaque area by image-pro plus software, n = 3-5. (**E-G**) The level of Aβ in the PFC and hippocampal tissue of AD mice, n = 5-6. Data are as mean ± SEM. *p < 0.05, **p < 0.01, ***p < 0.001. *P* values are calculated by one-way ANOVA with Tukey's post-hoc test.

Next, we investigated whether miR-206-3p inhibition could ameliorate learning and memory deficits in AD mice. After bilateral hippocampus injection, miR-206-3p antagomirs significantly reduced miR-206-3p in the prefrontal cortex and hippocampus of AD mice induced by Aβ brain injection (Figure 6A-C). In the MWM test, AD mice treated with miR-206-3p antagomirs showed shorter escape latency (Figure 6D and 6F) and path length (Figure S8A) than antagomir NC-treated mice. In the spatial probe test, miR-206-3p antagomirs significantly increased the number of crossing platform and the time spent in the target quadrant (Figure 6E and 6G-H). In the Y-maze test, AD mice injected with miR-206-3p antagomirs exhibited a higher percentage of spontaneous alternation than AD mice treated with antagomir negative control (Figure 6I). There was no significant correlation between the percentage of spontaneous alternation and total arm entries or travel distance in movement (Figure S8B-E). Immunoblotting results showed that miR-206-3p antagomirs increased PSD95, GAP43, and SYN levels in the prefrontal cortex (Figure 6J-M) and hippocampus (Figure 6N-Q). The area of Aβ plaques in CA1, CA3, and DG regions of the hippocampus was decreased as detected by immunohistochemistry (Figure 7A-D). Immunoblotting illustrated that miR-206-3p depletion resulted in reducing the Aβ burden both in the prefrontal cortex and hippocampus of AD mice (Figure 7E-G). miR-206-3p antagomirs effectively enhanced learning and memory abilities, alleviated Aβ burden, and promoted neurite growth in AD mice consistent with the effects of AEVs-_Aβ+H_ treatment, suggesting that the downregulated level of miR-206-3p in AEVs-_Aβ+H_ plays a key role in alleviating neurodegeneration of AD.

### miR-206-3p negatively regulates BDNF to alleviate AD phenotype

**Figure 8:**
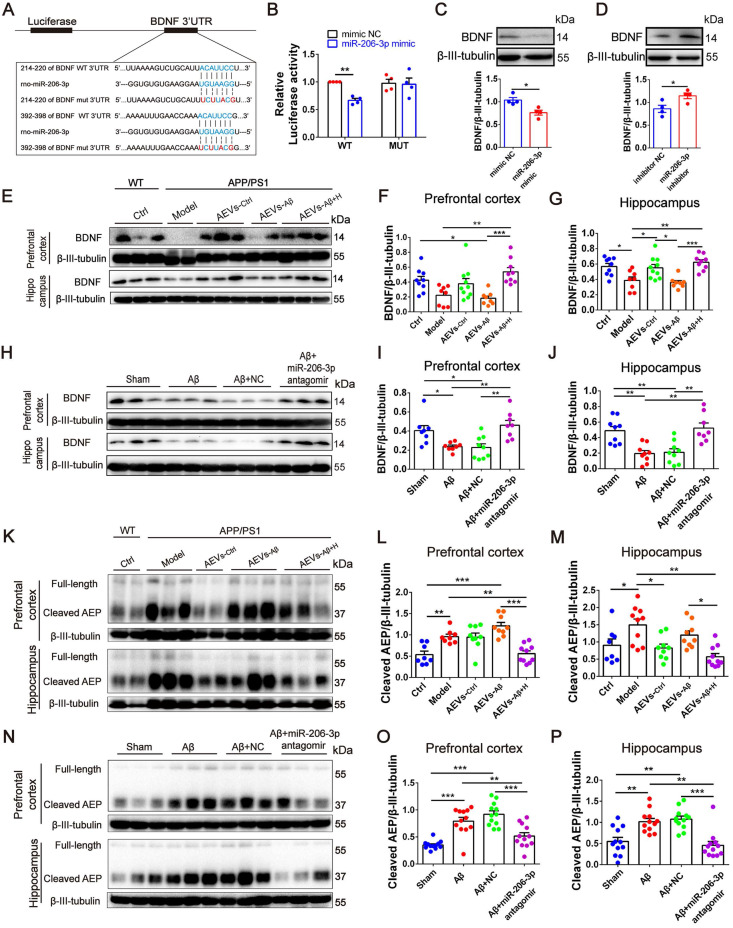
** AEVs-_Aβ+H_ and miR-206-3p antagomirs increase BDNF levels.** (**A**) Potential binding sites in 3'UTR of BDNF. (**B**) Identification of miR-206-3p potential binding sites to BDNF 3'UTR performed by luciferase reporter assay, n = 4. (**C-D**) The expression of BDNF in rat primary cortical neurons transfected with miR-206-3p mimics and inhibitors, n = 4. (**E-G**) BDNF expression in the PFC and hippocampus of APP/PS1 with intranasal administration of AEVs, n = 8-10. (**H-J**) The expression of BDNF in the PFC and hippocampus of AD mice with hippocampal injection of miR-206-3p antagomirs and antagomir NC, n = 8/9. (**K-M**) The level of AEP (full-length and cleaved AEP) in the PFC and hippocampus of APP/PS1 mice with intranasal administration of AEVs, n = 8-10. (**N-P**) The level of AEP (full-length and cleaved AEP) in the PFC and hippocampus of AD mice with hippocampal injection of miR-206-3p antagomirs, n = 11/12. Data are mean ± SEM. *p < 0.05, **p < 0.01, ***p < 0.001. *P* values are calculated by one-way ANOVA with Tukey's post-hoc test.

Using miRWalk and TargetScan databases, BDNF was predicted to be one of the potential target genes of miR-206-3p. BDNF plays a key role in axonal guidance and neurotrophic signaling pathway enriched by KEGG analysis. We next confirmed the targeted binding of miR-206-3p to BDNF. The 3'UTR region of BDNF containing miR-206-3p binding sites (wild-type, wt) and mutated sequence (mutant-type, mut) was inserted into the psiCHECK-2 plasmid (Figure 8A). The luciferase reporter assay was conducted by co-transfecting HEK293T cells with recombinant psiCHECK-2 vectors and miR-206-3p mimics. miR-206-3p mimics suppressed the relative luciferase activity in HEK293T cells transfected with the wild-type vector but not by the mutant type (Figure 8B). These data demonstrated that miR-206-3p bound to BDNF 3'-UTR sequences and inhibited BDNF expression. BDNF was downregulated by the high level of miR-206-3p in APP/PS1 mice compared with the normal control mice, while BDNF was upregulated in the prefrontal cortex and hippocampus of APP/PS1 mice with intranasal administration of AEVs-_Aβ+H_ and AEVs-_Ctrl_ (Figure 8E-G). The BDNF level in brain was also elevated by AEVs-_Aβ+H_ in Aβ brain-injected mice (Figure S9A-E). The expression of BDNF was downregulated or upregulated *in vitro* in neurons treated with miR-206-3p mimics or inhibitors, respectively (Figure 8C-D). Consistent with the *in vitro* results, miR-206-3p antagomirs significantly increased BDNF expression in the prefrontal cortex and hippocampus of Aβ brain-injected mice compared with no treatment (Figure 8H-J). These data demonstrated that BDNF was negatively regulated in neurons by miR-206-3p. Because miR-206-3p mimics inhibited the expression of PSD95, GAP43, and SYN, we examined whether miR-206-3p could target these genes via direct binding. Luciferase reporter assay was employed to detect the binding, and the results showed that PSD95, GAP43, and SYN were not direct targets of miR-206-3p (Figure S10).

It is noteworthy that the Aβ burden was significantly reduced accompanied by the increased BDNF level in the AD mice treated with AEVs-_Aβ+H_ or miR-206-3p antagomirs. The studies in AD models suggest that δ-secretase (AEP) is a key BDNF target in Aβ production and senile plaque formation 31, 32. Thus, we hypothesized that miR-206-3p deficiency in AEVs-_Aβ+H_ reduced AEP activation by increasing BDNF level, resulting in decreased Aβ. Interestingly, the cleaved AEP (active AEP) level was decreased in AEVs-_Aβ+H_ and AEVs-_Ctrl_-treated but not in AEVs-_Aβ_-treated APP/PS1 mice (Figure 8K-M), consistent with the upregulation of BDNF and reduction of Aβ. The same results were observed in Aβ mice injected with AEVs-_Aβ+H_ into the brain (Figure S9F-H). Also, miR-206-3p antagomirs reduced the level of cleaved AEP in the prefrontal cortex and hippocampus of AD mice (Figure 8N-P), further demonstrating that miR-206-3p/BDNF-regulated AEP played a critical role in Aβ clearance for AEVs-_Aβ+H_ treatment. However, AEVs-_Aβ+H_ and miR-206-3p antagomirs did not affect the expression of other APP cleaving enzymes, such as ADAM10, BACE1, and Aβ clearance protein IDE (Figure S11).

**Figure 9:**
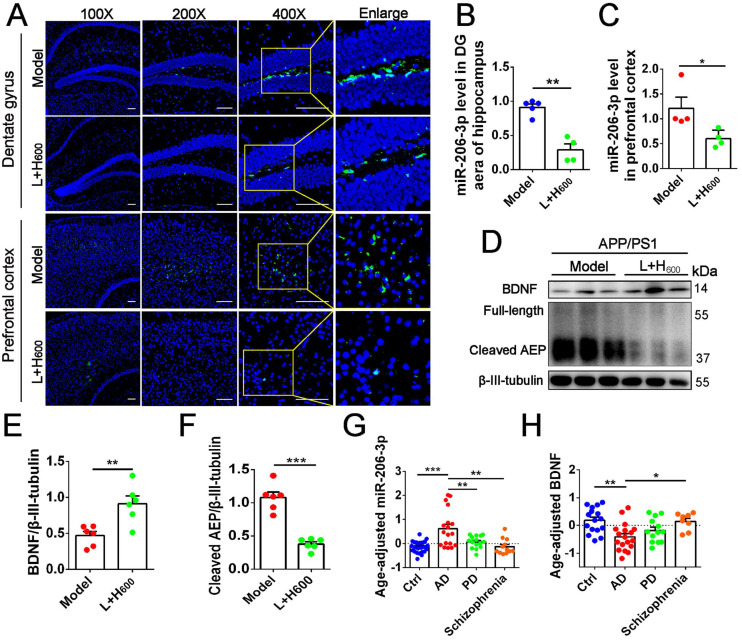
** haFGF_14-154_ inhibits AEP activity *via* miR-206-3p/BDNF pathway in the brain of APP/PS1 mice.** (**A-C**) miR-206-3p level in the PFC and hippocampal DG region of APP/PS1 mice with intranasal administration of 600 µg/kg haFGF_14-154_-loaded cationic liposomes (L+H_600_) or 0.9% saline (vehicle), measured by fluorescence *in situ* hybridization (FISH), n = 4/5. (**D-F**) The expression of BDNF and AEP (full-length and cleaved AEP) in the PFC of APP/PS1 mice with intranasal administration of L+H_600_ or vehicle, n = 6. The plasma levels of miR-206-3p **(G)** and BDNF **(H)** in patients with Alzheimer's disease (AD), PD and Schizophrienia detected by qPCR. *P* values are calculated by age-adjusted ANCOVA model with Tukey's post-hoc test. n = 25/16, 18/18, 15/13 and 12/8 for normal control and patients with AD, PD and Schizophrienia in the experiments of qPCR and ELISA, respectively. Data are mean ± SEM. *p < 0.05, **p < 0.01, ***p < 0.001. *P* values are calculated by one-way ANOVA with Tukey's post-hoc test and student's *t-test*.

We previously demonstrated that intranasal administration of Tat-haFGF-loaded cationic liposomes attenuated behavioral deficits in APP/PS1 mice 23. In this study, we found that Tat-haFGF treatment (L+H_600_) significantly downregulated miR-206-3p in the prefrontal cortex and hippocampus (Figure 9A-C) while BDNF was significantly increased compared with APP/PS1 mice (Figures 9D and E). Likewise, cleaved AEP was significantly decreased in the prefrontal cortex of APP/PS1 mice by intranasal administration of Tat-haFGF (Figures 9D and F). To assess the association between miR-206-3p and clinical diagnosis of AD, the miR-206-3p level was measured in the plasma of patients with AD, PD, and schizophrenia. After adjusting for age, we found that the plasma miR-206-3p level increased by 5.78-fold, and plasma BDNF concentration decreased by 3.22-fold in patients with AD compared with healthy individuals. Interestingly, AD individuals showed high miR-206-3p and low BDNF levels in plasma. On the contrary, changes observed in PD or schizophrenia patients were not significant (Figure 9G-H). Also, no correlation was observed between plasma miR-206-3p or BDNF and gender in the cohort (Figure S12).

## Discussion

Astrocytes, a large population of cells in the CNS, play important roles in neuronal development, growth, and synaptic formation 33-35. Astrocytes become over-activated in the pathological process of AD. The abnormal production of inflammatory cytokines and transmitters derived from continuously activated astrocytes could trigger neurodegenerative processes 36-40. The neuroprotective and neurodegenerative functions of astrocytes largely depend on the secreted molecules released into the extracellular space 41. As the major mediator in astrocyte-neuron communication, EVs display attractive characteristics for treating neurological diseases, including biological barrier permeability, high stability, and low toxicity. Increasing evidence suggests that EV cargoes, such as miRNA, mRNA, and proteins, are involved in neurodegenerative disorders. For example, IL-6, IL-1β, and TNF-α levels in AEVs are higher in AD patients than in healthy individuals 42. EVs deliver toxic cargoes throughout the brain, including tau, Aβ, α-synuclein, SOD1, and apolipoprotein D 43-45.

Neuron-derived AEVs and EVs (NEVs) can be isolated in the plasma of AD patients. These AD AEVs induce neurotoxicity with complement membrane attack complex (MAC) accumulation, membrane disruption, and necroptosis activation 30. The aFGF content in serum and cerebrospinal fluid is increased in AD patients 21. Interestingly, aFGF attenuates the activation of astrocytes 24, suggesting that aFGF modifies AEV cargoes involved in AD progression. It is intriguing whether AD AEVs stimulated by aFGF can attenuate the Alzheimer phenotype and whether there are some as yet unidentified messenger molecules involved in neuroprotection. As a noninvasive therapy, intranasal administration allows direct delivery of drugs to the brain through the olfactory nerve and olfactory bulb without crossing the blood-brain barrier (BBB), and reduces systemic exposure and side effects to treat diseases of the CNS 46. For these advantages, we intranasally administered different AEVs to AD mice. We found that following intranasal administration, AEVs entered the brain, and AEVs-_Aβ+H_ attenuated the cognitive behavior deficits and Aβ burden, improving neurite growth similar to AEVs produced in a normal situation (AEVs-_Ctrl_).

We sequenced AEV sRNAs to investigate the mechanisms underlying AEVs_-Aβ+H_ alleviation of AD syndromes in mice and found high expression of miR-206-3p in AEVs_-Aβ_. Consistent with this, miR-206-3p was increased in the hippocampus and cortex of AD model mice, including APP/PS1 mice and those in which Aβ_1-42_ was injected into the brain. The intracellular level of miR-206-3p was 4.47-fold higher in astrocytes than in neurons damaged by Aβ_1-42_.

AEVs are believed to be the main carrier of miR-206-3p in the AD brain. Previously, a study demonstrated that AEVs could overcome the BBB and spread into the plasma of AD patients 30. Therefore, we decided to investigate the miR-206-3p level in the plasma of patients suffering from common neurological disorders, such as AD, PD, and schizophrenia. Interestingly, of the three groups of patients studied, AD individuals had high plasma levels of miR-206-3p, whereas no significant change was observed in PD or schizophrenia patients. This finding showed that miR-206-3p plasma level could serve as a potential biomarker for AD diagnosis. Consistent with our results, Lee *et, al.* 47 reported that miR-206 was upregulated in the brain of APP/PS1 mice and the temporal cortex of AD patients, and miR-206 antagomirs improved the memory function of APP/PS1 mice. Remarkably, the miR-206-3p level was suppressed by aFGF in AEVs_-Aβ+H,_ and miR-206-3p antagomirs showed the same improvement in AEVs_-Aβ+H_ treated-AD mice. These studies demonstrated that reduction of miR-206-3p plays a key role in alleviating neurodegeneration of AD and that miR-206-3p might be a therapeutic target for AD treatment.

Next, we explored the mechanisms underlying the attenuation of AD neurodegeneration. We demonstrated that BDNF was negatively regulated by miR-206-3p and upregulated by AEVs_-Aβ+H_ and miR-206-3p antagomirs in the *in vitro* AD model and *in vivo*. Many studies have shown that BDNF expedites neurite elongation, increases synaptic density, and promotes the synaptic formation of neurons 48-50. As a major pathological hallmark in AD, the self-polymerizing Aβ accumulation is caused by its overproduction or reduced clearance 51. Although it has been reported that EVs derived from various cells (astrocytes, bone marrow mesenchymal stem cells, adipose-derived mesenchymal stem cells, etc) reduce Aβ deposition in the AD brain, the mechanism(s) still needs to be elucidated 16, 29, 52. In the present study, both AEVs-_Aβ+H_ and miR-206-3p antagomirs promoted BDNF levels in the AD brain while the Aβ burden was reduced, suggesting that BDNF might be involved in Aβ deposition. Recent reports show that AEP plays a key role in AD pathogenesis by cleaving APP 53. BDNF mimics alleviated Aβ plaque deposition in 5×FAD mice by repressing AEP activation 31, but deprivation of BDNF increased the Aβ level through upregulation of cleaved-AEP *via* the JAK2/STAT3/C/EBPβ/AEP pathway in wild-type mice 32. We also found that AEVs-_Aβ+H_ and miR-206-3p antagomir inhibited AEP activation to alleviate Aβ plaque deposition, while other APP-cleaving enzymes, such as ADAM10, BACE1, and Aβ clearance proteins IDE, had no noticeable difference in their expression level.

In conclusion, aFGF-stimulated AEVs provided favorable neuroprotection in AD pathology by enhancing neurite growth and reducing Aβ loading in neurons *in vitro*. AEVs-_Aβ+H_ ameliorated cognitive behavior deficits, promoted synaptic plasticity, and alleviated brain Aβ burden in AD mice. Furthermore, aFGF stimulation modified the cargoes in AEVs, the most significant of which being the down-regulation of miR-206-3p, which was specifically high in the plasma of AD mice and patients. Also, miR-206-3p antagomir reversed the Alzheimer phenotype in AD mice. BDNF gene was negatively regulated by miR-206-3p and upregulated by AEVs_-Aβ+H_ in AD mice. AEVs_-Aβ+H_ inhibited AEP activation *via* the miR-206-3p/BDNF axis to alleviate the Aβ burden in the AD brain. These findings highlight the significant roles of aFGF in modifying AEV cargoes and miR-206-3p as a potential biomarker for AD diagnosis and therapy.

## Supplementary Material

Supplementary figures.

## Figures and Tables

**Table 1:** Clinical information of the human plasma samples

Health Control			Alzheimer's disease (AD)			Parkinson's disease (PD)			Schizophrenia		
diagnosis	Age	gender	diagnosis	Age	gender	diagnosis	Age	gender	diagnosis	Age	gender
Normal-1	51	female	AD-1	57	female	PD-1	46	male	Sch-1	55	female
Normal-2	51	female	AD-2	55	male	PD-2	84	male	Sch-2	55	female
Normal-3	73	male	AD-3	82	male	PD-3	65	female	Sch-3	49	female
Normal-4	57	male	AD-4	80	male	PD-4	80	male	Sch-4	48	female
Normal-5	62	male	AD-5	59	male	PD-5	71	female	Sch-5	72	female
Normal-6	71	female	AD-6	81	female	PD-6	70	female	Sch-6	48	male
Normal-7	61	female	AD-7	66	female	PD-7	58	male	Sch-7	58	female
Normal-8	63	female	AD-8	84	male	PD-8	80	male	Sch-8	49	male
Normal-9	53	female	AD-9	78	male	PD-9	75	male	Sch-9	53	male
Normal-10	60	female	AD-10	71	male	PD-10	81	male	Sch-10	55	female
Normal-11	70	female	AD-11	86	male	PD-11	67	female	Sch-11	51	male
Normal-12	58	male	AD-12	63	male	PD-12	67	male	Sch-12	52	female
Normal-13	51	male	AD-13	86	male	PD-13	81	male			
Normal-14	68	male	AD-14	59	female	PD-14	58	male			
Normal-15	58	male	AD-15	85	female	PD-15	57	female			
Normal-16	69	female	AD-16	67	female						
Normal-17	52	female	AD-17	58	female						
Normal-18	50	female	AD-18	65	female						
Normal-19	57	female									
Normal-20	38	male									
Normal-21	45	male									
Normal-22	59	male									
Normal-23	71	male									
Normal-24	48	female									
Normal-25	64	female									

## Abbreviations

AD: Alzheimer's disease
Aβ: amyloid-beta peptide
AEVs: EVs derived from astrocyte
AEVs-_Ctrl_: EVs derived from normal astrocytes
AEVs-_Aβ_: EVs derived from astrocytes treated with Aβ
AEVs-_Aβ+H_: EVs derived from damaged astrocytes treated with Aβ and haFGF_14-154_
ADAM10: a disintegrin and metalloprotease 10
ALS: amyotrophic lateral sclerosis
AEP: Asparagine endopeptidase
BACE1: beta-site APP Converting Enzyme 1
BBB: blood-brain barrier
BDNF: brain-derived neurotrophic factor
CNS: central nervous system
DG: dentate gyrus
EVs: Extracellular versicles
GAP43: growth-associated protein 43
haFGF: human acidic fibroblast growth factor
IDE: insulin-degrading enzyme
MWM: Morris water maze
NFTs: neurofibrillary tangles
NEVs: EVs derived from neurons
PSD95: post-synaptic density-95
RPM: reads per million clean tags
SYN: Synaptophysin
