# Supplementary material for: Extracellular vesicles derived from astrocyte-treated with haFGF14-154 attenuate Alzheimer phenotype in AD mice
Source: Theranostics. 2022 May 9;12(8):3862–81. doi: 10.7150/thno.70951 (PMC9131260; doi:10.7150/thno.70951)
Supplement: Supplementary file 1 — Supplementary figures. [file thnov12p3862s1.pdf]

Extracellular vesicles derived from astrocyte-treated with haFGF$_{14-154}$ attenuate Alzheimer phenotype in AD mice

Dong Peng[a,b], Youjin Wang[a,b], Yuanjie Xiao[a,b], Mengyuan Peng[a,b], Wanwen Mai[a,b], Bo Hu[d], Yanbin Jia[e], Hongxia Chen[a,b,c], Yan Yang[a,b,c], Qi Xiang[a,b,c], Zhijian Su[a,b,c], Qihao Zhang[a,b,c*], Yadong Huang[a,b,c*]

* Correspondence: Qihao Zhang, Department of Cell Biology & Institute of Biomedicine, College of Life Science and Technology, Jinan University, Guangzhou 510632, China. E-mail: tqhzhang@jnu.edu.cn. Yadong Huang, Cell Biology Department & Institute of Biomedicine, College of Life Science and Technology, Jinan University, Guangzhou 510632, China. E-mail: tydhuang@jnu.edu.cn

Supplemental Figure legends

Figure S1 Isolation and characterize of AEVs. (**A**) A representative immunofluorescence staining with the antibody of glial fibrillary acidic protein (GFAP, Green, Scale: 50 μm). (**B**) A schematic diagram displayed the treatment for astrocytes with $A\beta_{1-42}$ and $haFGF_{14-154}$. (**C-E**) Representative immunoblots (**C**) and quantification (**D-E**) of exosome biomarker proteins (CD9, CD63, and clathrin) in astrocytes and AEVs lysates, n = 4. Representative Endoplasmic reticulum biomarker calnexin used as negative control. (**F**) Representative particle size and distribution of AEVs observed by NanoSight Tracking Analysis (NTA). (**G**) Representative Atomic force microscopy (AFM) image of AEVs. $^{*}p < 0.05$, $^{**}p < 0.01$. *P* values are calculated by one-way ANOVA with Tukey's post-hoc test.

Figure S2 Tracking AEVs labeled by PKH26. (**A**) Representative images of AEVs labeled by PKH26 (red) in primary cortical neurons stained by green as $MAP2^{+}$ cells (Scale bars: 20 μm). Neurons and AEVs were co-cultured for 0.5 h. (**B**) The schematic diagram of intranasal administration of AEVs (60 μg) labeled by PKH26 (red). (**C**) The distribution of AEVs was detected in olfactory bulb (OB) and entorhinal cortex (EC) of APP/PS1 mice in 6 h after single intranasal administration. The negative control mice were given volume-matched PBS.

Figure S3 Supplementary data for APP/PS1 mice in Y-maze and MWM test. In Y-maze test, total arm entries (**A**), total traveled distance (**B**), the correlation between total arm entries and spontaneous alternation (**C**), the correlation between total traveled distance and spontaneous alternation (**D**) in APP/PS1 mice, n = 6-8. (**E**) The movement distance of escape latency for APP/PS1 mice in MWM test, n = 8. $^{*}p < 0.05$, $^{**}p < 0.01$. *P* values are calculated by two-way ANOVA with Tukey's post-hoc test.

Figure S4 AEVs-$_{A\beta+H}$ ameliorate learning and memory deficits in AD mice with brain injection of Aβ$_{1\text{-}42}$. (**A**) Experimental protocols of intranasal administration and behavior test of AD mice with hippocampal injection of Aβ$_{1\text{-}42}$. (**B**) Spontaneous alternation in Y-maze, n = 8-10. (**C-F**) In Y-maze test, total arm entries (**C**), total traveled distance (**D**), the correlation between total arm entries and spontaneous alternation (**E**), the correlation between total traveled distance and spontaneous alternation (**F**) in AD mice with Aβ brain injection, n = 8-10. (**G-I**) Navigation test in MWM, n = 7/8. Representative movement tracks of escape latency on day 5 in navigation test (**G**) and the escape latency (**H**, **I**). (**J**) The movement distance of escape latency for AD mice with Aβ brain injection in MWM test, n = 7/8. (**K-M**) Spatial probe test was performed 24 h after the navigation test. Representative movement tracks in spatial probe test (**K**), numbers of crossing the removed platform area (**L**) and time spend in target quadrant (**M**) in spatial probe test of MWM, n = 7/8. **p* < 0.05, ***p* < 0.01, ****p* < 0.001. *P* values are calculated by two-way ANOVA with Tukey's post-hoc test.

Figure S5 AEVs-$_{A\beta+H}$ enhance expression of synaptic proteins in AD mice with brain injection of Aβ$_{1\text{-}42}$. (A, E) Representative immunoblotting images of PSD95, GAP43 and SYN in the PFC (**A**) and hippocampus (**E**) of AD mice. The quantified expression of the above proteins in the PFC (**B-D**) and hippocampus (**F-H**), n = 5-6. Data are mean ± SEM. ***p* < 0.01, ****p* < 0.001. *P* values are calculated by one-way ANOVA with Tukey's post-hoc test.

Figure S6 AEVs-$_{A\beta+H}$ reduce Aβ deposition in AD mice with brain injection of Aβ$_{1\text{-}42}$. (**A**) Aβ plaque in the hippocampal DG, CA1 and CA3 regions of AD mice detected by immunochemistry. (**B-D**) The area of Aβ plaque analyzed by image-pro plus software, n = 4-6. (**E-G**) The level of Aβ in the PFC and hippocampus detected

by Western blot, n = 4-6. Data are mean ± SEM. *$p < 0.05$, **$p < 0.01$. *P* values are calculated by one-way ANOVA with Tukey's post-hoc test.

Figure S7 Analysis of miRNAs in AEVs by small RNA sequence. (**A**) Representative density distribution map of miRNAs in different AEVs. Light orange indicates AEVs-$_{Ctrl}$, light green indicates AEVs-$_{A\beta}$ and light gray indicates AEVs-$_{A\beta+H}$. (**B-D**) Volcanic maps exhibit statistically differentially expressed miRNAs between the AEVs-$_{Ctrl}$ and AEVs-$_{A\beta}$ (**B**), AEVs-$_{A\beta}$ and AEVs-$_{A\beta+H}$ (**C**), AEVs-$_{Ctrl}$ and AEVs-$_{A\beta+H}$ (**D**). The red and green dots indicate miRNAs that are up-regulated and down-regulated respectively, and the gray dots indicate miRNAs that are no statistical differences.

Figure S8 Supplementary data for Aβ brain injection mice treated with miR-206-3p antagomir in Y-maze and MWM test. (**A**) Representative the movement distance of escape latency for Aβ brain injection mice treated with miR-206-3p antagomir in MWM test, n = 9-12. In Y-maze test, total arm entries (**B**), total traveled distance (**C**), the correlation between total arm entries and spontaneous alternation (**D**), the correlation between total traveled distance and spontaneous alternation (**E**) in Aβ brain injection mice treated with miR-206-3p antagomir, n = 9-12. Data are mean ± SEM. *$p < 0.05$. *P* values are calculated by one-way ANOVA with Tukey's post-hoc test and two-way ANOVA with Tukey's post-hoc test.

Figure S9 AEVs-$_{A\beta+H}$ increases BDNF levels and inhibits the AEP activity in the brain of AD mice. (**A-C**) BDNF expression in PFC and hippocampus of AD mice with $A\beta_{1-42}$ brain injection, n = 9-11. (**D-E**) BDNF level in the PFC and hippocampal tissues detected by Elisa, n = 4. (**F**) Representative immunoblotting images of AEP (full-length and cleaved AEP) in the PFC and hippocampus. (**G-H**) The quantified expression of AEP, n = 7-8. Data are mean ± SEM. *$p < 0.05$, **$p < 0.01$, ***$p <$

0.001. *P* values are calculated by one-way ANOVA with Tukey's post-hoc test.

Figure S10 No direct target relationship between miR-206-3p and the synaptic proteins (PSD95, GAP43 and SYN). Dual-luciferase reporter assay has showed that miR-206-3p does not directly bind to the 3′UTR of PSD95 (**A**), GAP43 (**B**) and SYN (**C**). Data are mean ± SEM. n = 3. *P* values are calculated by one-way ANOVA with Tukey's post-hoc test (n.s.: not significant).

Figure S11 AEVs-$_{\mathbf{A\beta+H}}$ and miR-206-3p antagomir have no effects on the expression of APP cleaving enzymes (ADAM10, BACE1) and Aβ clearance proteins (IDE). Representative the immunoblots (**A**) and quantified expression of ADAM10, BACE1 and IDE in PFC (**B-D**) and hippocampus (**E-H**) of APP/PS1 mice with intranasal administration of AEVs, n = 5-6. (**I-O**) The expression of ADAM10, BACE1 and IDE in the PFC (**I**, **J-L**) and hippocampus (**I**, **M-O**) of Aβ brain injection mice with intranasal administration of AEVs, n = 5-6. The representative immunoblots (**P**) and quantified expression of ADAM10, BACE1, and IDE in the PFC (**Q-S**) and hippocampus (**T-V**) of AD mice injected with miR-206-3p antagomir, n = 9. Data are mean ± SEM. $^{*}p < 0.05$ and $^{**}p < 0.01$. *P* values are calculated by one-way ANOVA with Tukey's post-hoc test.

Figure S12 The correlation between plasma miR-206-3p or BDNF and gender in the whole cohort. The correlation between plasma miR-206-3p and gender in the healthy individuals (**A**, Ctrl), AD patients (**B**), PD patients (**C**) and Schizophrenia patients (**D**), n = 25, 18, 15 and 12 for normal control and patients with AD, PD and Schizophrienia respectively. The correlation between plasma BDNF and gender in the healthy individuals (**E**, Ctrl), AD patients (**F**), PD patients (**G**) and Schizophrenia patients (**H**), n = 16, 18, 13 and 8 for normal control and patients with AD, PD and Schizophrienia respectively. Data are calculated by Pearson correlations and best

fitted with linear regression curve.

Figure S1

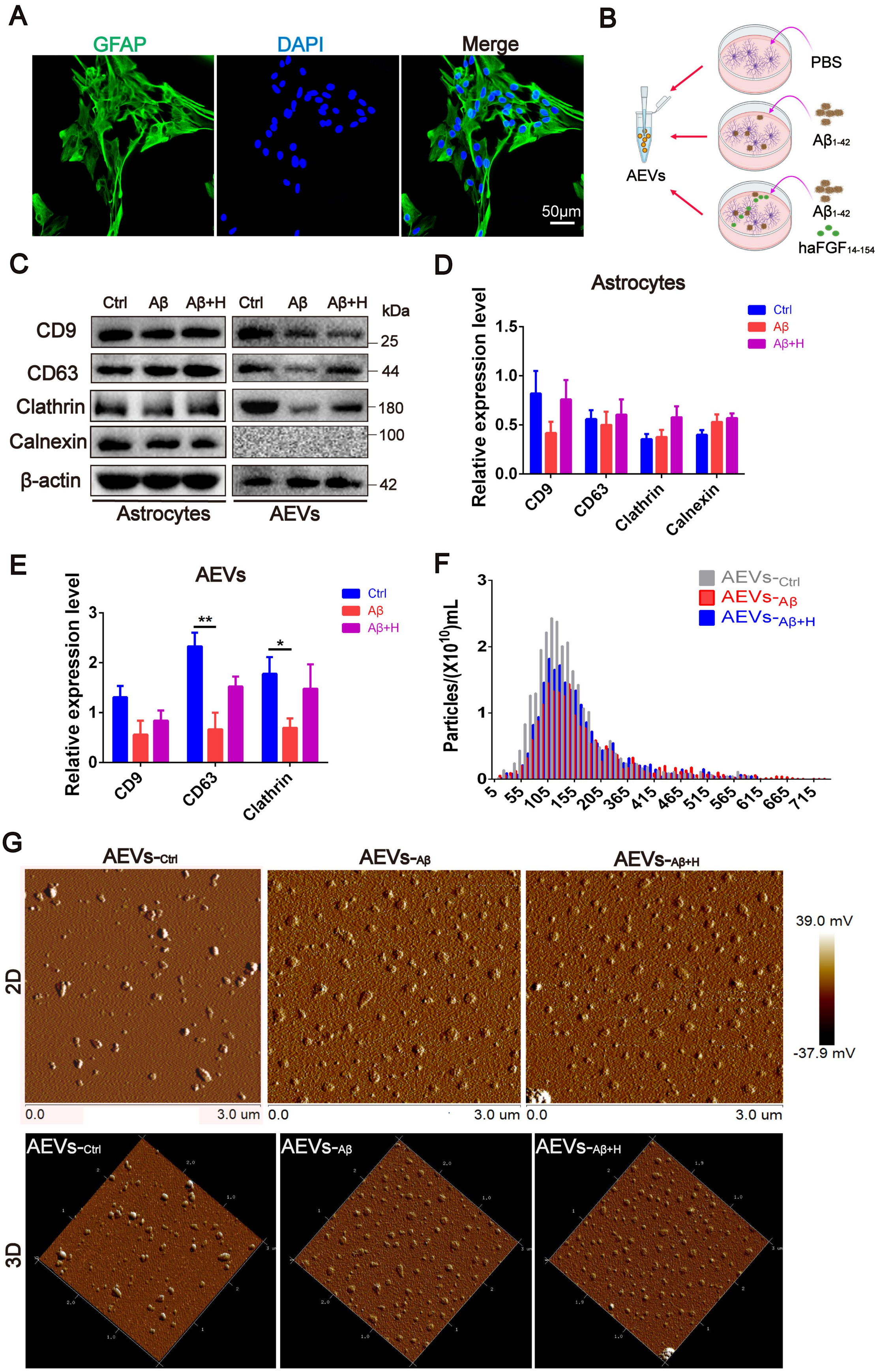

A
GFAP
DAPI
Merge
50μm
B
PBS
Aβ1-42
Aβ1-42
haFGF14-154
AEVs
C
Ctrl
Aβ
Aβ+H
Ctrl
Aβ
Aβ+H
kDa
CD9
CD63
Clathrin
Calnexin
β-actin
25
44
180
100
42
Astrocytes
AEVs
D
Astrocytes
Relative expression level
Ctrl
Aβ
Aβ+H
CD9
CD63
Clathrin
Calnexin
E
AEVs
Relative expression level
Ctrl
Aβ
Aβ+H
CD9
CD63
Clathrin
F
AEVs-Ctrl
AEVs-Aβ
AEVs-Aβ+H
Particles/(X10^10)mL
G
AEVs-Ctrl
AEVs-Aβ
AEVs-Aβ+H
2D
39.0 mV
-37.9 mV
0.0
3.0 um
3D

Figure S2

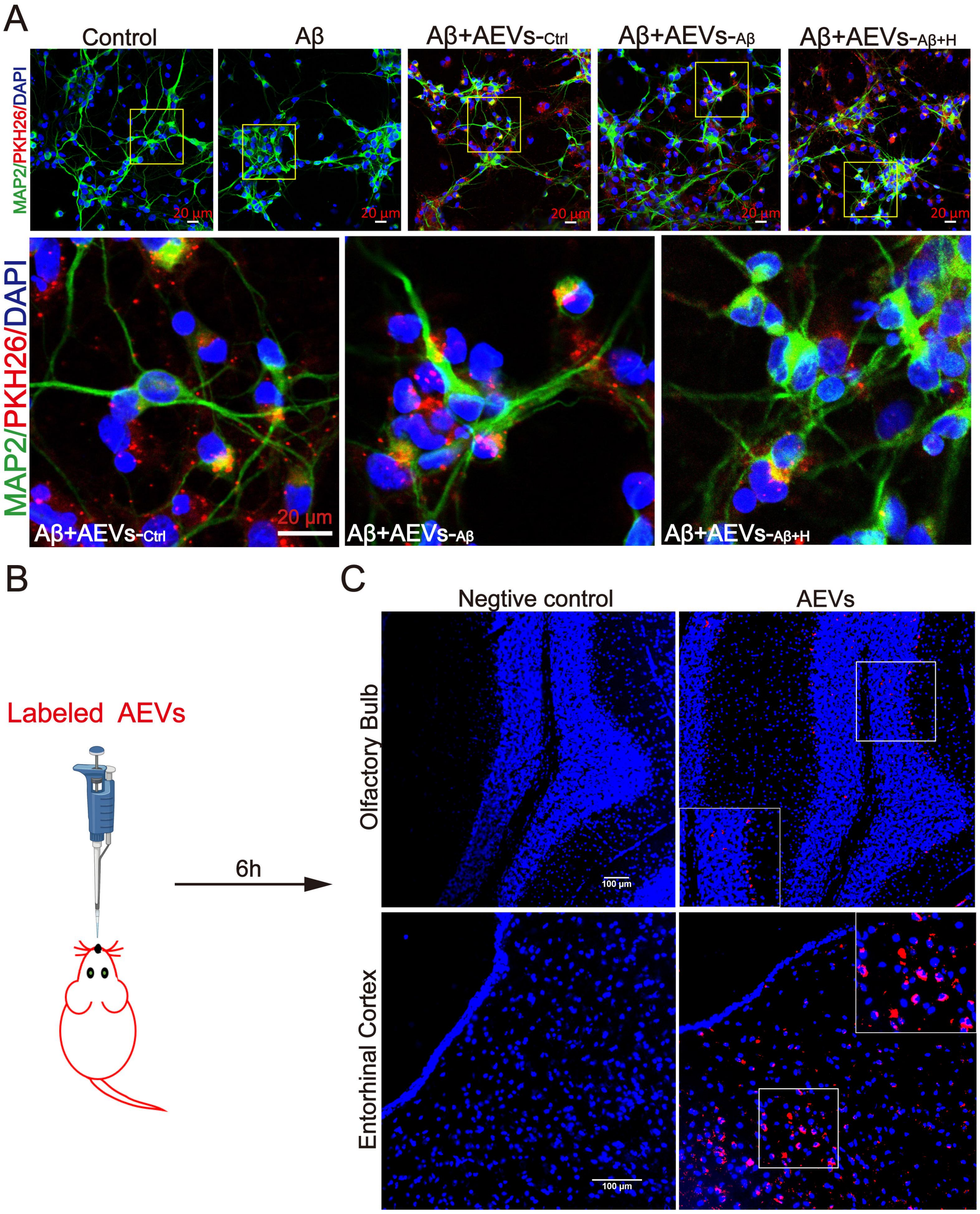
A
Control
Aβ
Aβ+AEVs-Ctrl
Aβ+AEVs-Aβ
Aβ+AEVs-Aβ+H
MAP2/PKH26/DAPI
20 μm
Aβ+AEVs-Ctrl
Aβ+AEVs-Aβ
Aβ+AEVs-Aβ+H
B
Labeled AEVs
6h
C
Negtive control
AEVs
Olfactory Bulb
Entorhinal Cortex
100 μm

Figure S3

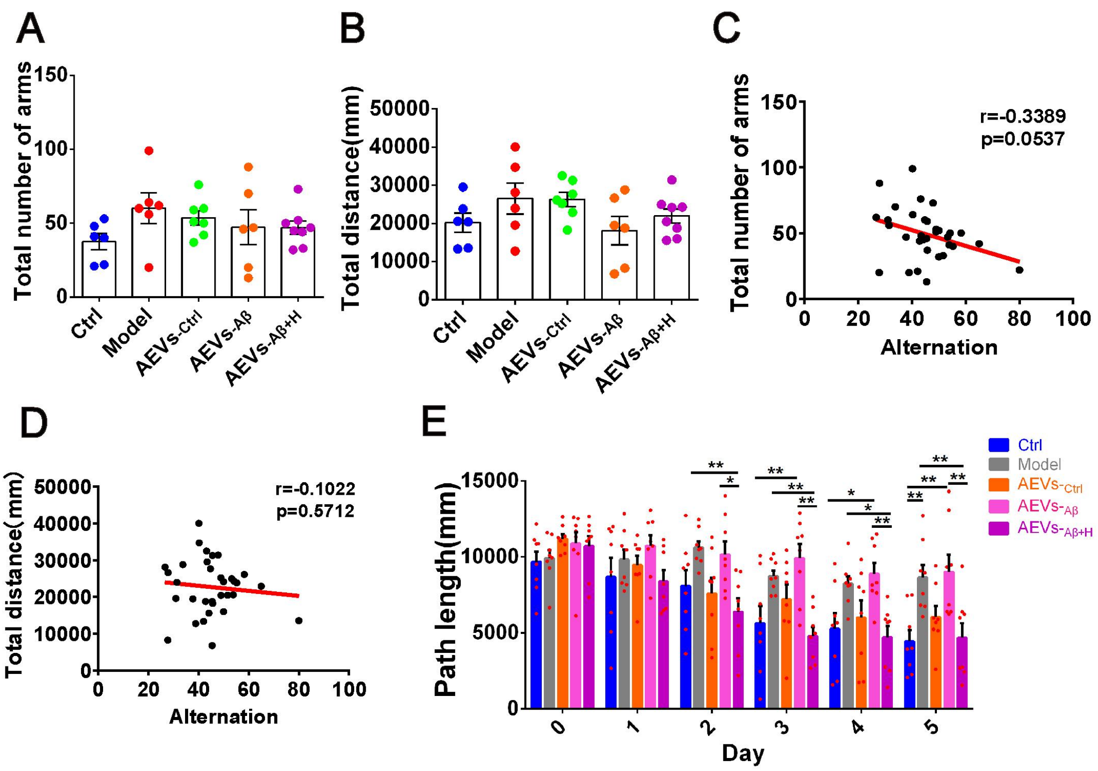

A
Total number of arms
Ctrl
Model
AEVs-Ctrl
AEVs-Aβ
AEVs-Aβ+H
B
Total distance(mm)
C
Total number of arms
Alternation
r=-0.3389
p=0.0537
D
Total distance(mm)
Alternation
r=-0.1022
p=0.5712
E
Path length(mm)
Day
Ctrl
Model
AEVs-Ctrl
AEVs-Aβ
AEVs-Aβ+H

Figure S4

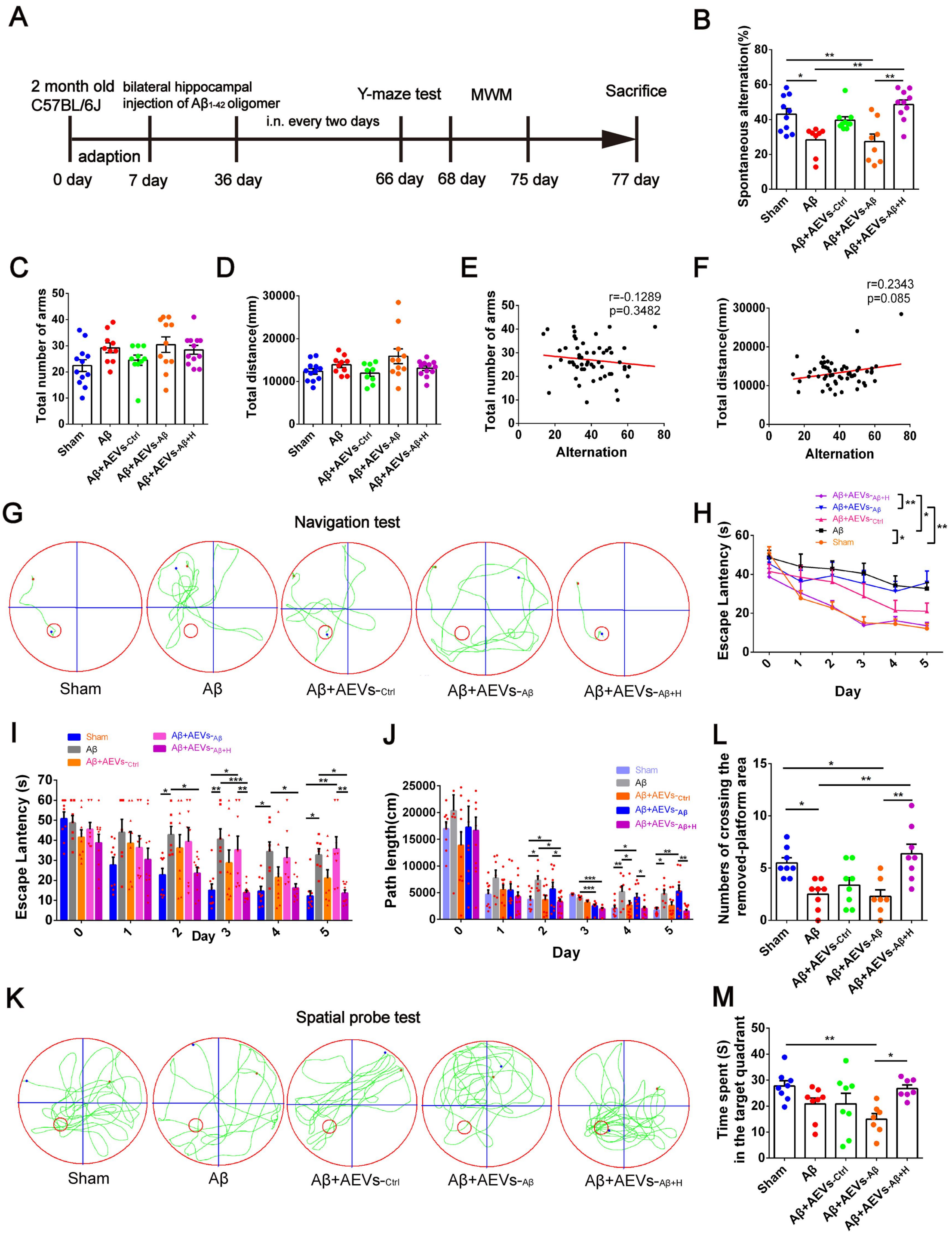
A
2 month old C57BL/6J
bilateral hippocampal injection of Aβ1-42 oligomer
i.n. every two days
Y-maze test
MWM
Sacrifice
adaption
0 day
7 day
36 day
66 day
68 day
75 day
77 day
B
Spontaneous alternation(%)
C
Total number of arms
D
Total distance(mm)
E
r=-0.1289
p=0.3482
Total number of arms
Alternation
F
r=0.2343
p=0.085
Total distance(mm)
Alternation
G
Navigation test
Sham
Aβ
Aβ+AEVs-Ctrl
Aβ+AEVs-Aβ
Aβ+AEVs-Aβ+H
H
Escape Lantency (s)
Day
I
Escape Lantency (s)
Day
J
Path length(cm)
Day
L
Numbers of crossing the removed-platform area
K
Spatial probe test
M
Time spent (S) in the target quadrant

Figure S5

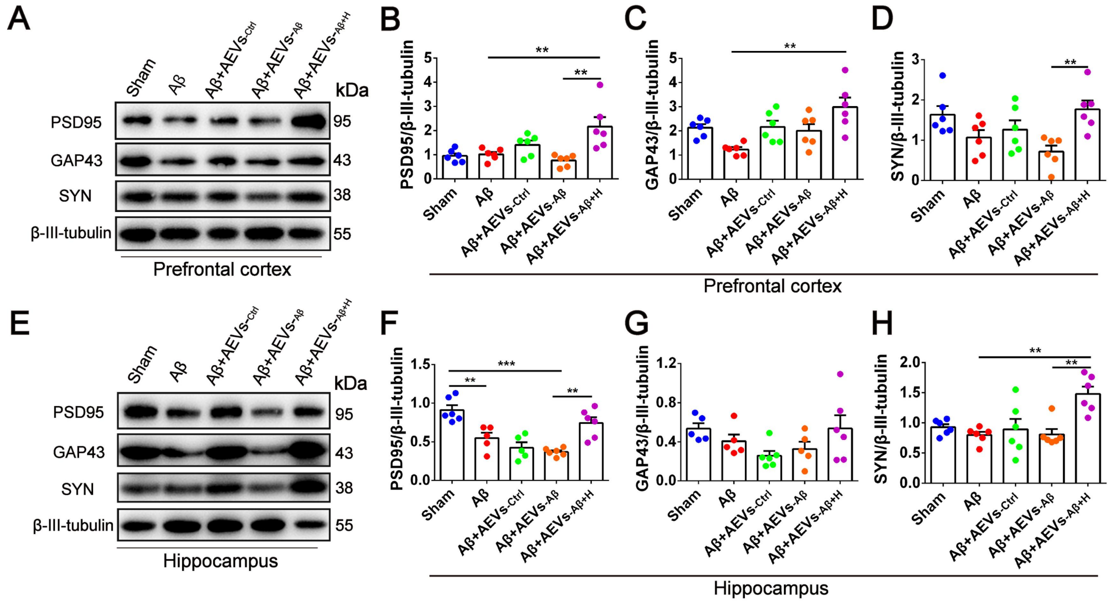

A
Sham
Aβ
Aβ+AEVs-Ctrl
Aβ+AEVs-Aβ
Aβ+AEVs-Aβ+H
kDa
PSD95 95
GAP43 43
SYN 38
β-III-tubulin 55
Prefrontal cortex
B
PSD95/β-III-tubulin
C
GAP43/β-III-tubulin
D
SYN/β-III-tubulin
Prefrontal cortex
E
Hippocampus
F
PSD95/β-III-tubulin
G
GAP43/β-III-tubulin
H
SYN/β-III-tubulin
Hippocampus

Figure S6

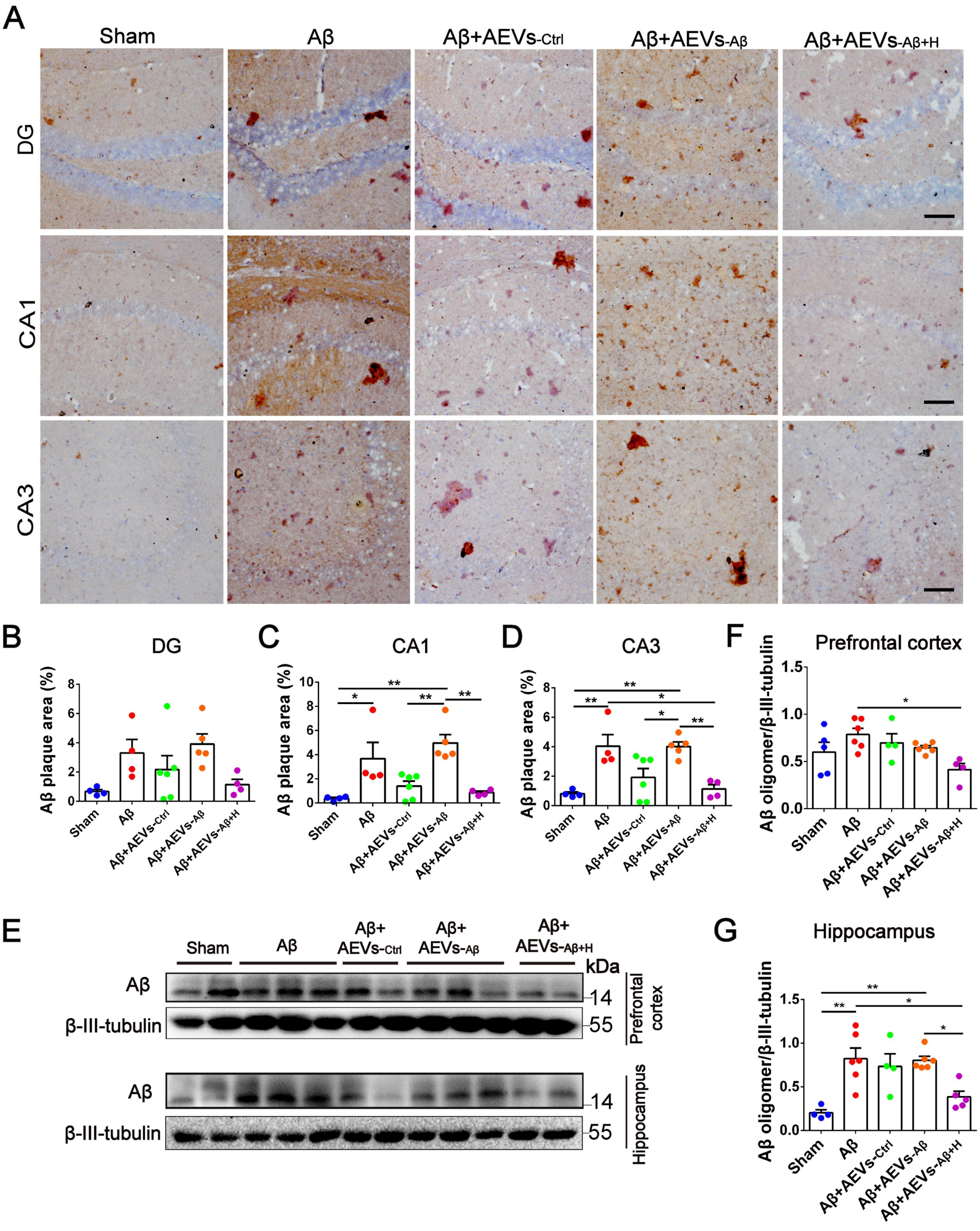
A
Sham
Aβ
Aβ+AEVs-Ctrl
Aβ+AEVs-Aβ
Aβ+AEVs-Aβ+H
DG
CA1
CA3
B
DG
Aβ plaque area (%)
C
CA1
D
CA3
F
Prefrontal cortex
Aβ oligomer/β-III-tubulin
E
Sham
Aβ
Aβ+ AEVs-Ctrl
Aβ+ AEVs-Aβ
Aβ+ AEVs-Aβ+H
kDa
Aβ
β-III-tubulin
14
55
Prefrontal cortex
Hippocampus
G
Hippocampus

Figure S7

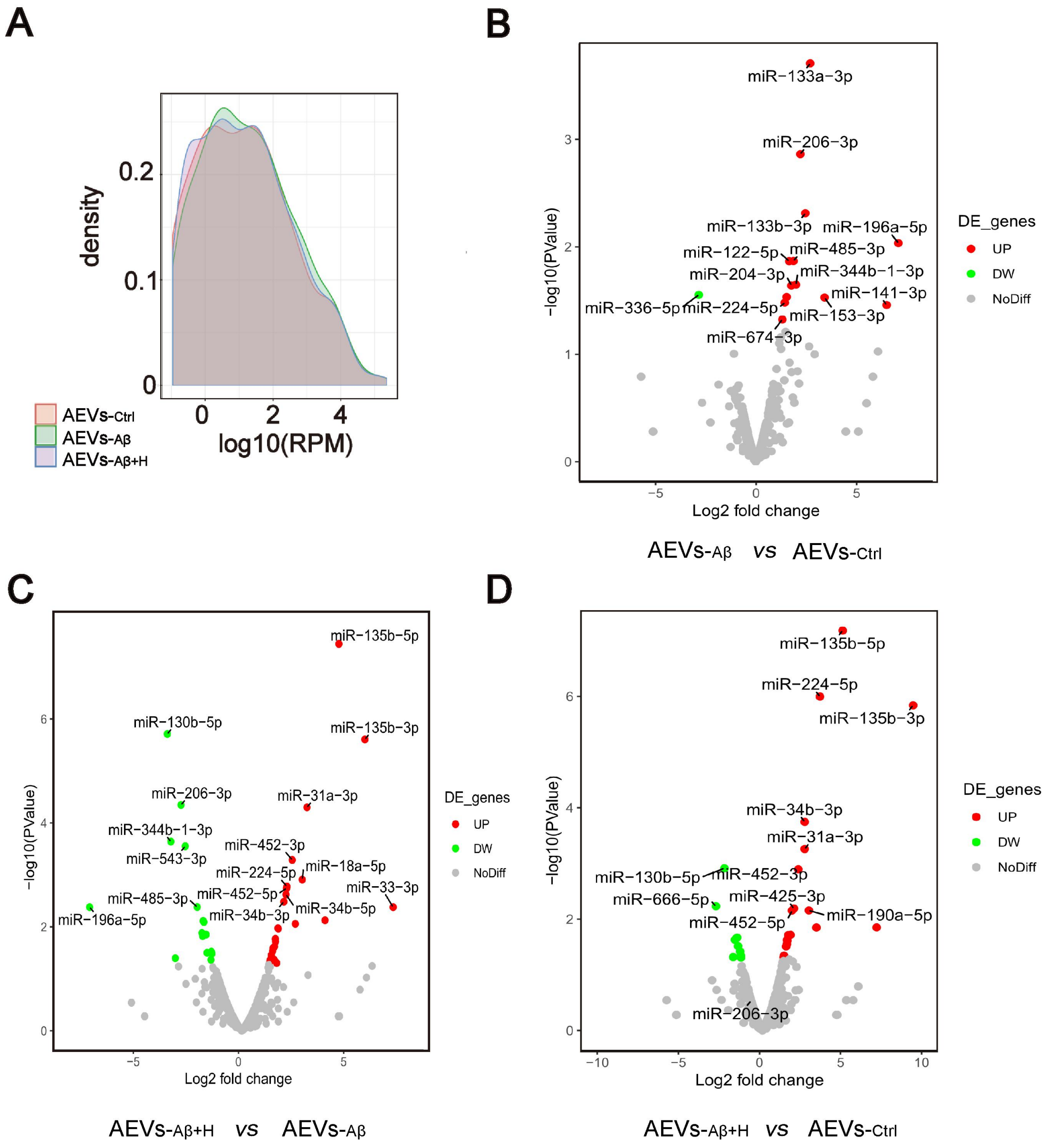

A
density
log10(RPM)
AEVs-Ctrl
AEVs-Aβ
AEVs-Aβ+H
B
miR−133a−3p
miR−206−3p
miR−133b−3p
miR−196a−5p
miR−122−5p
miR−485−3p
miR−204−3p
miR−344b−1−3p
miR−141−3p
miR−336−5p
miR−224−5p
miR−153−3p
miR−674−3p
−log10(PValue)
Log2 fold change
DE_genes
UP
DW
NoDiff
AEVs-Aβ vs AEVs-Ctrl
C
miR−135b−5p
miR−130b−5p
miR−135b−3p
miR−206−3p
miR−31a−3p
miR−344b−1−3p
miR−452−3p
miR−543−3p
miR−18a−5p
miR−224−5p
miR−33−3p
miR−485−3p
miR−452−5p
miR−34b−5p
miR−196a−5p
miR−34b−3p
−log10(PValue)
Log2 fold change
DE_genes
UP
DW
NoDiff
AEVs-Aβ+H vs AEVs-Aβ
D
miR−135b−5p
miR−224−5p
miR−135b−3p
miR−34b−3p
miR−31a−3p
miR−130b−5p
miR−452−3p
miR−666−5p
miR−425−3p
miR−190a−5p
miR−452−5p
miR−206−3p
−log10(PValue)
Log2 fold change
DE_genes
UP
DW
NoDiff
AEVs-Aβ+H vs AEVs-Ctrl

Figure S8

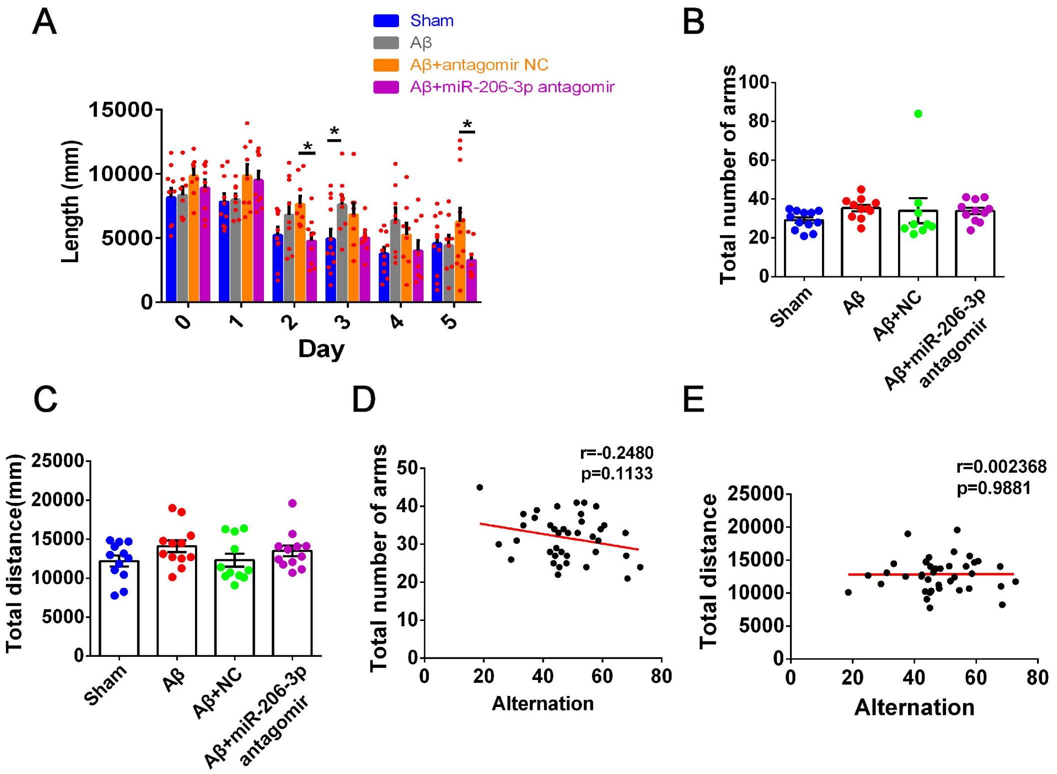

A
Sham
Aβ
Aβ+antagomir NC
Aβ+miR-206-3p antagomir
Length (mm)
15000
10000
5000
0
0
1
2
3
4
5
Day
*
*
*
B
Total number of arms
100
80
60
40
20
0
Sham
Aβ
Aβ+NC
Aβ+miR-206-3p antagomir
C
Total distance(mm)
25000
20000
15000
10000
5000
0
Sham
Aβ
Aβ+NC
Aβ+miR-206-3p antagomir
D
r=-0.2480
p=0.1133
Total number of arms
50
40
30
20
10
0
0
20
40
60
80
Alternation
E
r=0.002368
p=0.9881
Total distance
25000
20000
15000
10000
5000
0
0
20
40
60
80
Alternation

Figure S9

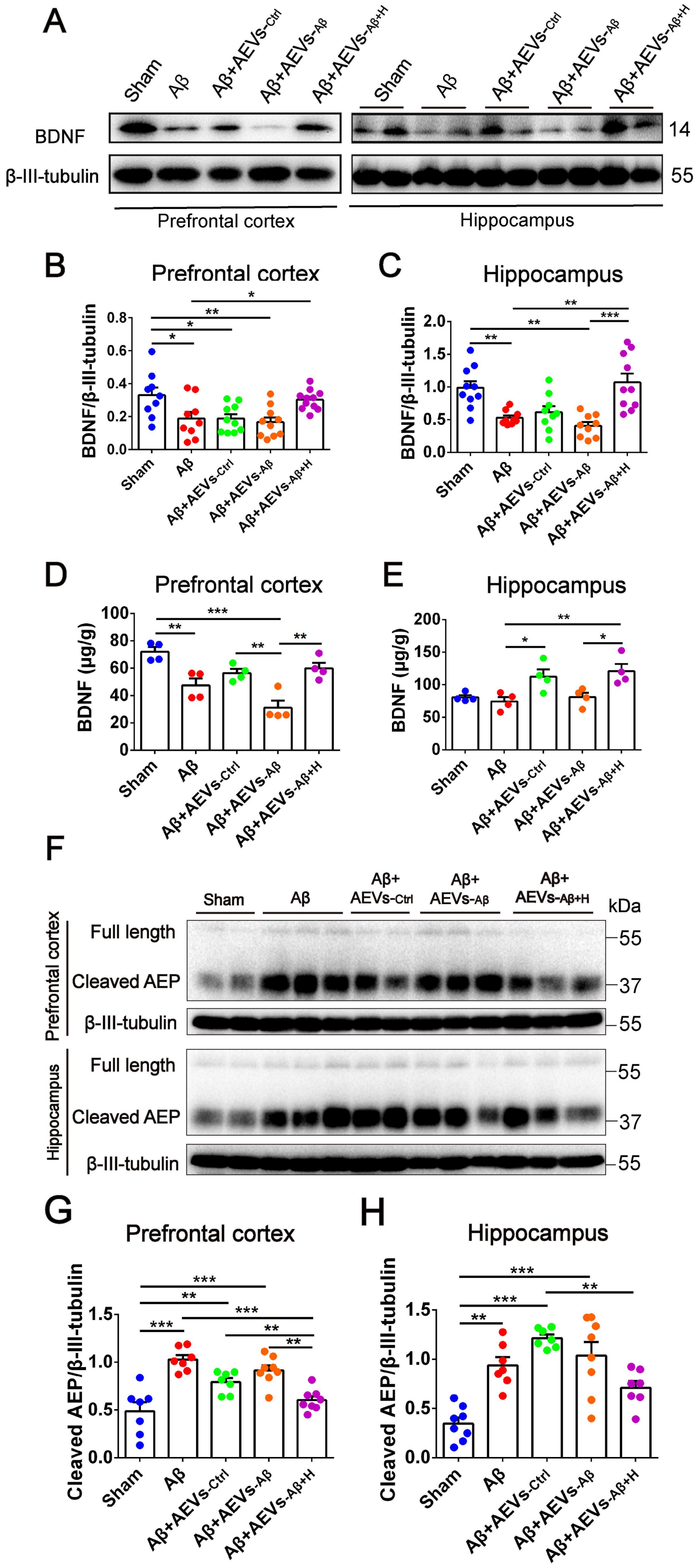

A
Sham
Aβ
Aβ+AEVs-Ctrl
Aβ+AEVs-Aβ
Aβ+AEVs-Aβ+H
BDNF
β-III-tubulin
14
55
Prefrontal cortex
Hippocampus
B
Prefrontal cortex
BDNF/β-III-tubulin
C
Hippocampus
D
Prefrontal cortex
BDNF (μg/g)
E
Hippocampus
F
Full length
Cleaved AEP
kDa
G
Prefrontal cortex
Cleaved AEP/β-III-tubulin
H
Hippocampus

Figure S10

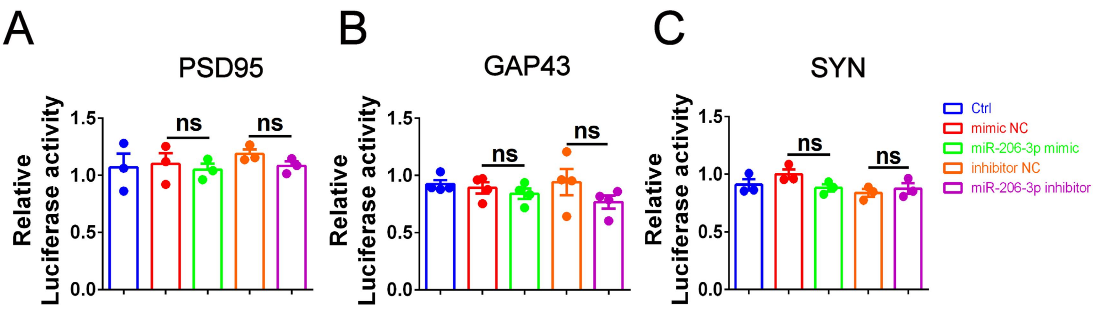

A
PSD95
B
GAP43
C
SYN
Relative Luciferase activity
1.5
1.0
0.5
0.0
ns
Ctrl
mimic NC
miR-206-3p mimic
inhibitor NC
miR-206-3p inhibitor

Figure S11

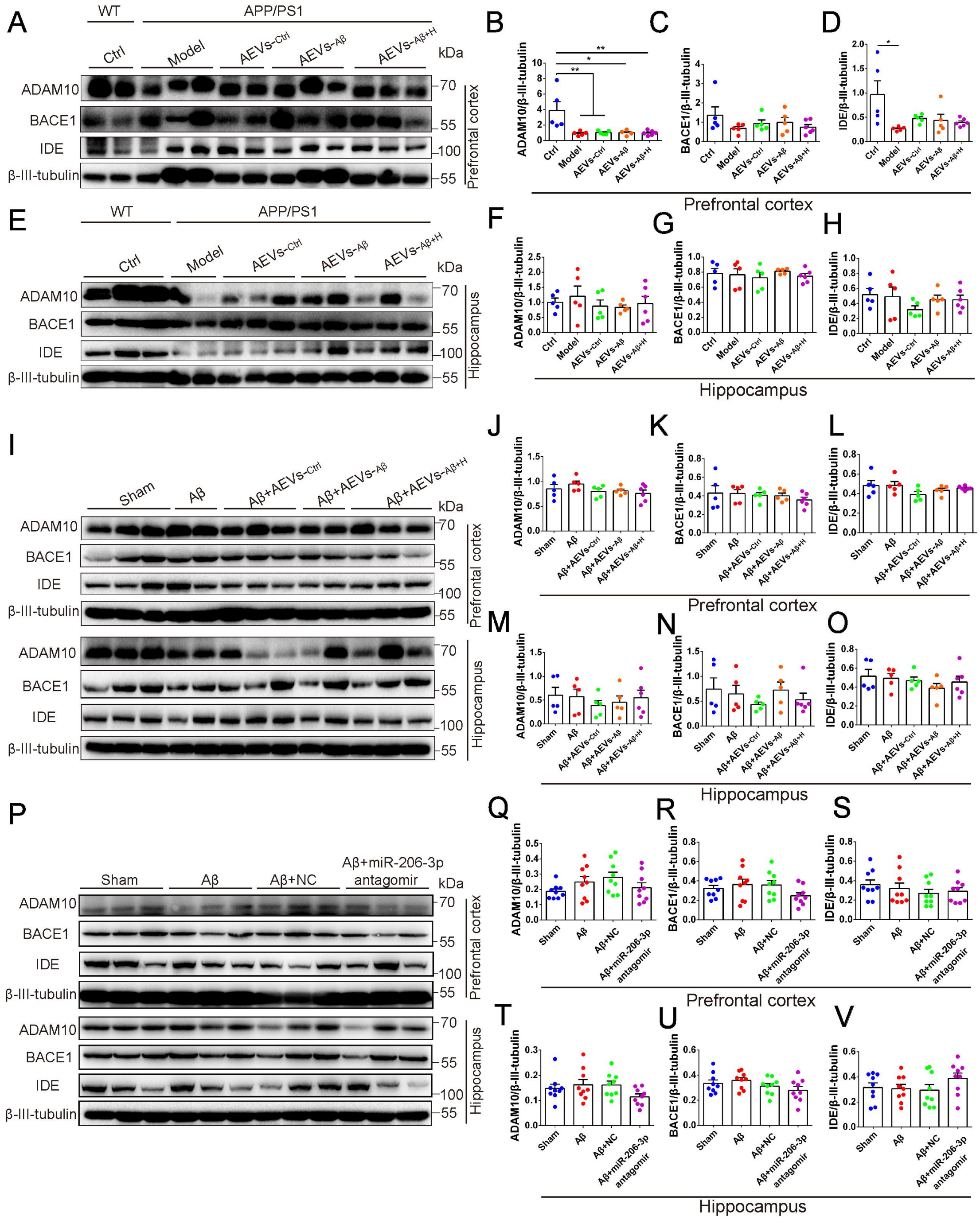
A
WT
APP/PS1
Ctrl
Model
AEVs-Ctrl
AEVs-Aβ
AEVs-Aβ+H
kDa
ADAM10
BACE1
IDE
β-III-tubulin
70
55
100
55
Prefrontal cortex
B
C
D
ADAM10/β-III-tubulin
BACE1/β-III-tubulin
IDE/β-III-tubulin
Prefrontal cortex
E
F
G
H
Hippocampus
I
Sham
Aβ
Aβ+AEVs-Ctrl
Aβ+AEVs-Aβ
Aβ+AEVs-Aβ+H
J
K
L
M
N
O
P
Aβ+NC
Aβ+miR-206-3p antagomir
Q
R
S
T
U
V

Figure S12

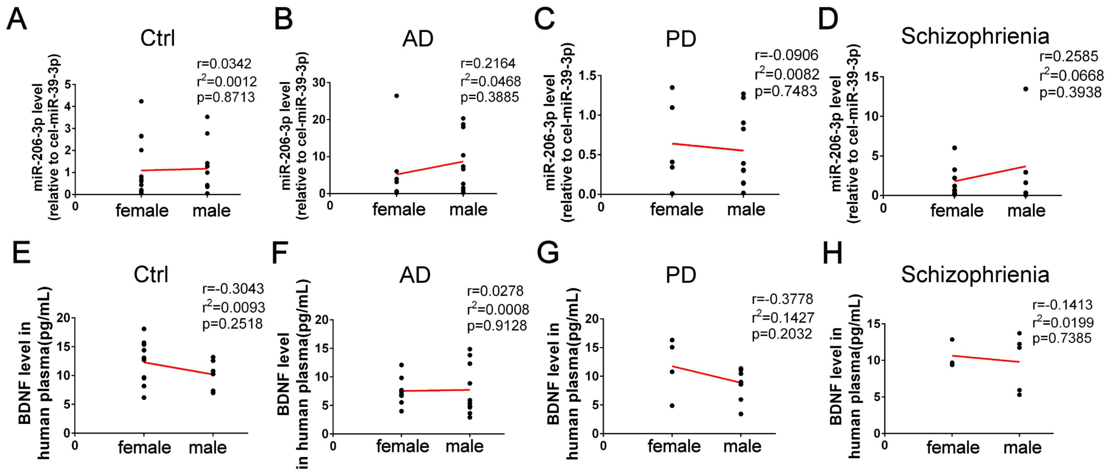

A
Ctrl
r=0.0342
r2=0.0012
p=0.8713
miR-206-3p level (relative to cel-miR-39-3p)
female
male
B
AD
r=0.2164
r2=0.0468
p=0.3885
C
PD
r=-0.0906
r2=0.0082
p=0.7483
D
Schizophrienia
r=0.2585
r2=0.0668
p=0.3938
E
Ctrl
r=-0.3043
r2=0.0093
p=0.2518
BDNF level in human plasma(pg/mL)
F
AD
r=0.0278
r2=0.0008
p=0.9128
G
PD
r=-0.3778
r2=0.1427
p=0.2032
H
Schizophrienia
r=-0.1413
r2=0.0199
p=0.7385
